# Assessment of Potential Exposure to Pregnancy-Contraindicated Medications Among Women of Reproductive Age in Japan: A Retrospective Database Study

**DOI:** 10.3390/pharmacy14020051

**Published:** 2026-03-20

**Authors:** Hiroyuki Ura, Noriko Matsuoka

**Affiliations:** 1 Department of Clinical Pharmacy, Shonan University of Medical Science, Yokohama 244-0806, Japan; 2 Department of Pharmacy Services, Nippon Medical School Musashi Kosugi Hospital, Kawasaki 211-8533, Japan

**Keywords:** drug prescribing patterns, women of reproductive age, potential exposure, preconception care, pregnancy contraindications, pharmacist intervention

## Abstract

Preconception care is globally recognized as essential for optimizing pregnancy outcomes; however, in Japan, comprehensive data on medication-related potential exposure to pregnancy-contraindicated medications among women of reproductive age remain limited. We conducted a retrospective cross-sectional descriptive study using data from Japan’s National Database of Health Insurance Claims (fiscal year 2022) to assess the potential exposure to pregnancy-contraindicated medications among women of reproductive age. Outpatient prescriptions for oral medications dispensed to women aged 15–49 years were analyzed. In total, 270 medications classified as contraindicated during pregnancy were identified, of which 75 were also contraindicated for women planning pregnancy. Of these, 58 active ingredients were restricted in both phases. Notably, 212 medications were uniquely contraindicated during pregnancy, highlighting the broader contraindication profiles during fetal development than during the preconception period. Despite these contraindications, high prescription volumes were observed for medications such as loxoprofen sodium hydrate, sodium valproate, and metformin hydrochloride among women of reproductive age. These findings illustrate a high baseline utilization of pregnancy-contraindicated medications among women of reproductive age. As most women in this demographic are neither pregnant nor actively planning conception, these volumes primarily reflect standard care rather than inappropriate prescribing. In conclusion, pharmacists serve as an important supplementary safety net by routinely confirming pregnancy status to prevent inadvertent exposure.

## 1. Introduction

The management of health conditions in women of reproductive age is a critical public health concern. Although pharmacotherapy is often necessary for managing chronic conditions, the use of certain medications may carry the risk of teratogenicity or adverse pregnancy outcomes [1]. The period of highest risk for drug-induced congenital malformations occurs during organogenesis (typically between 4 and 10 weeks of gestation), a time when many women may not yet be aware of their pregnancy [2]. Given that a substantial proportion of pregnancies are unplanned, ensuring medication assessment of potential exposure to pregnancy-contraindicated medications is relevant not only for confirmed pregnancies but for all women of childbearing potential. Recent large-scale birth cohort studies in Japan have demonstrated high baseline medication utilization among reproductive-age women. For instance, approximately 49.0% to 75.3% of pregnant women reported using medications (excluding vitamins and supplements) during the 12 months preceding pregnancy diagnosis [3,4]. These findings highlight that medication exposure before pregnancy recognition is highly prevalent in Japan, urgently necessitating population-level assessments of potential exposure.

Preconception care (PCC) aims to identify and modify biomedical, behavioral, and social risks to a woman’s health or pregnancy outcomes through prevention and management [5]. A key component of PCC is the assessment of medication use to prevent inadvertent exposure to teratogenic agents. Certain medications, such as valproic acid, are known to pose serious risks to the fetus [6]. However, despite safety warnings, real-world prescribing patterns often demonstrate continued use of high-risk medications among women of childbearing potential.

In Japan, although detailed pregnancy contraindication classifications for medications exist, regulatory labels often lag behind emerging clinical evidence, creating discrepancies that complicate clinical decision-making. Furthermore, comprehensive data on the scale of potential exposure—defined as the prescription of pregnancy-contraindicated medications to women of reproductive age who may conceive—remain limited. Previous studies have largely focused on specific disease cohorts or single institutions. Therefore, the primary objective of this study was to assess the potential exposure of women of reproductive age to pregnancy-contraindicated medications in Japan. We analyzed the National Database of Health Insurance Claims and Specific Health Checkups of Japan (NDB) Open Data, which covers nearly the entire Japanese population [7]. Unlike studies tracking individual pregnancy outcomes, this study utilizes aggregated prescription volumes to highlight the public health magnitude of prescribing pregnancy-contraindicated medications to women aged 15–49 years.

## 2. Materials and Methods

### 2.1. Data Collection

This was a retrospective cross-sectional descriptive study based on aggregated national claims data. We used the Strengthening the Reporting of Observational Studies in Epidemiology (STROBE) reporting guideline [8] to draft this manuscript, and the STROBE reporting checklist [9] when editing, included in Supplementary Table S1. Data from the NDB Open Data Japan for fiscal year (FY) 2022 (1 April 2022 to 31 March 2023) were obtained from the Ministry of Health, Labour and Welfare website [7]. The NDB Open Data Japan includes comprehensive coverage of 98.8% of medical claims from hospitals and clinics and 99.8% of dispensing claims from pharmacies as of March 2023 [10]. For dispensing claims from pharmacies, available variables include drug product names, therapeutic categories based on Japanese standard treatment codes, and total drug price calculation units (e.g., tablets, grams, milliliters), all grouped by sex and five-year age categories.

### 2.2. Target Population and Definitions

We extracted data for all oral medications prescribed to women aged 15–49 years, a group defined in this study as women of reproductive age. Additionally, to contextualize the prescribing patterns, we also extracted the total prescription volumes for the general population (all sexes and age groups) from the same NDB Open Data. As the NDB Open Data comprises aggregated volumes without individual patient identifiers or clinical diagnoses, pregnancy status or pregnancy intention could not be determined. Therefore, this study evaluates potential exposure based on the volume of medications prescribed to this demographic, rather than confirmed exposure during pregnancy. Data for other routes of administration (e.g., suppositories, injections) were excluded because they were not comprehensively available. All data were collected using a standardized extraction form and recorded in a Microsoft Excel spreadsheet, as previously described [11,12].

### 2.3. Medication Classification

All identified oral medications were consolidated into distinct active ingredients based on their therapeutic category codes. Each medication was classified into three categories using the latest package insert search system provided by the Pharmaceuticals and Medical Devices Agency [13]. Based on the description in the Pregnant Women section, ingredients were classified as follows:

1. Pregnancy-contraindicated: Medications explicitly prohibited for use in pregnant women or women who may be pregnant.
2. Use if benefit outweighs risk: Medications permitted only when the therapeutic benefits are judged to outweigh the potential risks.
3. No cautionary statement: Medications with no specific warnings regarding pregnancy.

Specific classification rules were applied to medications with multiple indications or specific warnings. Diabetes medications stating “Do not administer to pregnant women or women who may be pregnant; use insulin preparations” were classified as pregnancy-contraindicated. Medications were also classified as contraindicated if their active ingredients had multiple approved indications within the same therapeutic category and at least one of these indications specified a contraindication for pregnant women. For example, although sodium valproate is contraindicated for pregnant women with migraine but considered beneficial for pregnant women with epilepsy, it was classified as contraindicated for pregnant women. For active ingredients with multiple approved indications across different therapeutic categories, the package insert descriptions for each indication were applied separately. For instance, zonisamide was classified as beneficial for epilepsy treatment (code: 113) but contraindicated for pregnant women when used for Parkinsonism (code: 116).

Similarly, in the preconception context, medications were classified as contraindicated if the package insert mandated contraception or prohibited use in women planning to conceive. To minimize classification bias, two independent reviewers (H.U. and N.M.) independently classified all medications and resolved discrepancies through discussion. A conservative classification approach was adopted: medications were classified as contraindicated if any approved indication carried a pregnancy contraindication.

### 2.4. Data Analysis and Statistical Analysis

The primary outcome measure was the prescription volume, calculated as the sum of drug price calculation units (prescription units). We acknowledged that this total volume represents a mix of different units (e.g., tablets, grams, milliliters). However, for protein and amino acid preparations (code: 325), packaging units (e.g., cans or packets) were used to avoid overestimating prescription volumes. A comprehensive analysis of prescription volumes across therapeutic codes focused on medications prescribed to women of reproductive age (15–49 years).

Data management and analysis were performed using Python (v. 3.10.12), NumPy (v. 1.26.4), and Pandas (v. 2.2.2). Data are presented as descriptive statistics (total prescription volumes and percentages). The overlap of contraindications was visualized using Venn diagrams, and the proportion of contraindicated ingredients across therapeutic categories was compared using heat maps.

### 2.5. Ethics Statement

This study was approved by the Ethics Committee of the Shonan University of Medical Sciences (Approval number: 24-023). As this study exclusively used publicly available, aggregated data from the NDB Open Data Japan, which contain no individual patient identifiers or personal health information, written informed consent was not required. All research procedures were conducted in accordance with institutional guidelines and regulatory standards for data security and confidentiality protection. No individual-level data were accessed, and there was no possibility of identifying individual patients from the dataset.

## 3. Results

### 3.1. Classification of Oral Medication

**Figure 1:**
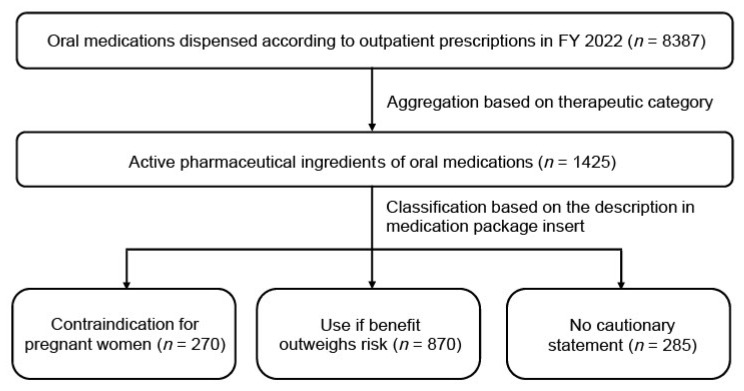
Flow diagram depicting the systematic classification of oral medications based on pregnancy-related package insert information in fiscal year 2022. Oral medications dispensed to women of reproductive age were consolidated into active pharmaceutical ingredients of oral medications and categorized based on the “Pregnant Women” section of the package inserts: contraindicated, use if benefit outweighs risk, and no cautionary statement.

A total of 8387 oral medications were identified from outpatient prescriptions in the NDB Open Data Japan database for FY 2022 [7]. These were consolidated into 1425 distinct active pharmaceutical ingredients of oral medications based on therapeutic category codes (Figure 1). As the NDB Open Data Japan provides pre-aggregated prescription volumes without individual patient-level records, assessment of individual-level missing data was not applicable; however, the database covers 98.8% of medical claims and 99.8% of dispensing claims nationwide, ensuring near-complete population coverage [10]. The medications were categorized into three groups according to the package insert information: contraindicated for pregnant women (*n* = 270, 18.9%), use if benefit outweighs risk for pregnant women (*n* = 870, 61.1%), and no cautionary statement for pregnant women (*n* = 285, 20.0%).

### 3.2. Distribution of Contraindicated Ingredients Across Therapeutic Categories

**Figure 2:**
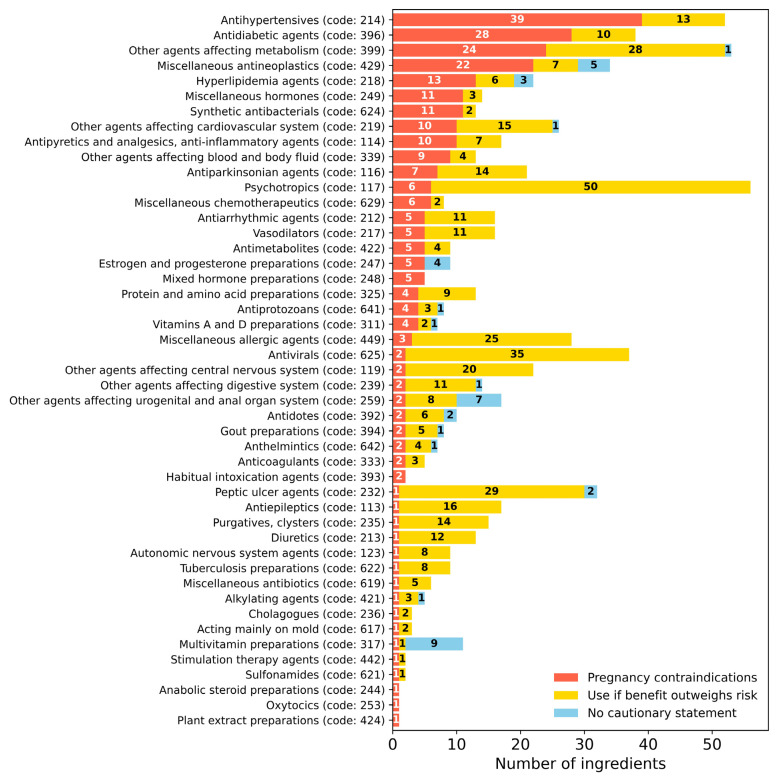
Distribution of pharmacological agents based on therapeutic categories and pregnancy-related package insert information. The horizontal bar graph illustrates the distribution of medications across therapeutic categories according to their package insert descriptions regarding pregnancy. Each bar segment represents: contraindications (orange), use if benefit outweighs risk (yellow), and no cautionary statement (blue). Therapeutic categories are arranged according to the standardized Japanese pharmaceutical classification codes.

Analysis of pregnancy-contraindicated medications across therapeutic categories revealed notable differences in their distribution patterns (Figure 2). Two therapeutic categories exhibited complete contraindications: mixed hormone preparations (code: 248) and habitual intoxication agents (code: 393), where all active ingredients were classified as contraindicated (5/5 and 2/2 ingredients, respectively). Categories with a high proportion of pregnancy-contraindicated medications included synthetic antibacterials (84.6%, 11/13 ingredients), miscellaneous hormones (78.6%, 11/14 ingredients), and antihypertensives (75.0%, 39/52 ingredients). The antidiabetic agents category also showed a substantial proportion of pregnancy-contraindicated medications, accounting for 73.7% (28/38 ingredients). In contrast, several therapeutic categories demonstrated minimal contraindications: peptic ulcer agents (3.1%, 1/32 ingredients), antivirals (5.4%, 2/37 ingredients), and antiepileptics (5.9%, 1/17 ingredients).

### 3.3. Prescription Volumes of Pregnancy-Contraindicated Medications in Women of Reproductive Age

We analyzed the prescription volumes of pregnancy-contraindicated medications specifically for women of reproductive age (15–49 years) to estimate potential exposure. Four therapeutic categories dominated the prescription volumes in this demographic: antipyretics, analgesics, and anti-inflammatory agents (code: 114); miscellaneous hormones (code: 249); antidiabetic agents (code: 396); and antiepileptics (code: 113). These categories accounted for approximately half of all potentially contraindicated prescription volumes in this age group (Table 1).

Certain therapeutic categories showed a high concentration of prescriptions among women of reproductive age relative to the general population. For example, oxytocics were frequently prescribed to women aged 15–49 years. Similarly, mixed hormone preparations (code: 248) accounted for 97.5% of all prescriptions in this demographic. However, because this category predominantly consists of combined oral contraceptives used to actively prevent pregnancy, this high volume reflects appropriate reproductive health management rather than unintended fetal exposure risk.

### 3.4. Analysis of Active Ingredients with Potential Exposure Risks

An analysis of the top 50 pregnancy-contraindicated medications prescribed to women of reproductive age revealed that these ingredients accounted for approximately 85% of all contraindicated prescriptions (Table 2). Loxoprofen sodium hydrate was the most frequently prescribed medication, accounting for 15.7% of all contraindicated prescription volumes, followed by sodium valproate (9.4%) and dienogest (8.8%).

Hormonal contraceptives, such as drospirenone/ethinylestradiol and norethisterone/ethinylestradiol, ranked fifth and sixth, comprising 4.6 and 3.9% of the contraindicated prescriptions, respectively. Other notable contraindicated prescriptions included lithium preparations (3.2%), rosuvastatin calcium (2.7%), and domperidone (2.7%). In the antidiabetic category, metformin hydrochloride accounted for 5.5% of contraindicated prescriptions, making it the leading contraindicated antidiabetic agent prescribed to women of reproductive age.

### 3.5. Comparison of Regulatory Contraindication Classifications Between Pregnancy and Preconception

**Figure 3:**
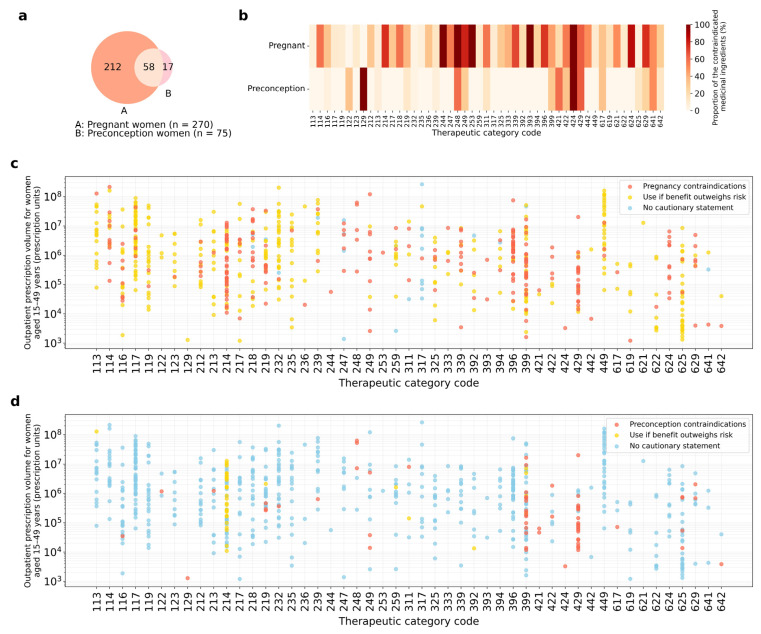
Visualization of medication regulatory contraindication patterns and prescription trends in reproductive healthcare. (**a**) Venn diagram illustrating the intersection between agents classified as contraindicated for pregnant women (*n* = 270) and those contraindicated for women planning pregnancy (*n* = 75). (**b**) Heat map showing the proportion of contraindicated agents within each therapeutic category. Darker red shades indicate higher proportions of contraindications. (**c**,**d**) Scatter plots demonstrating the relationship between prescription volumes (y-axis, presented on a logarithmic scale representing total prescription units) and therapeutic categories (x-axis) for women of reproductive age (15–49 years). While the data points (prescription volumes) are identical in both plots, the color coding differs to reflect the specific classifications for each period: (**c**) pregnancy regulatory classifications; (**d**) preconception regulatory classifications. Orange, pregnancy- or preconception-contraindicated medications; yellow, use if benefit outweighs risk; blue, no cautionary statement.

A comprehensive analysis of 1425 active ingredients revealed a marked disparity in medication regulatory contraindication patterns between the pregnancy and preconception periods. During pregnancy, 270 ingredients (18.9%) were classified as contraindicated, whereas only 75 ingredients (5.3%) were contraindicated during the preconception period (Figure 3a). Further analysis using Venn diagrams revealed an asymmetric pattern in the overlap of contraindications. Among the 75 agents contraindicated in women planning pregnancy, 58 (77.3%) were also contraindicated during pregnancy. However, of the 270 agents contraindicated during pregnancy, the majority (*n* = 212, 78.5%) were labeled as pregnancy-specific restrictions, with no specific contraindications for women planning pregnancy.

Analysis of the therapeutic categories revealed distinct patterns of contraindications between pregnancy and preconception periods. Hormonal preparations (code: 24x) showed a high contraindication rate for pregnant women (75.9%, 22/29 agents; Figure 2); however, only 24.1% of these medications were contraindicated for women planning pregnancy (Figure 3b–d). Mixed hormone preparations (code: 248) also displayed a distinctive pattern: all low-dose oral contraceptives were classified as contraindicated for women planning pregnancy, except for the estradiol/levonorgestrel combination for emergency contraception and the norgestrel/ethinylestradiol combination for infertility treatment (Table S2).

Analysis of other therapeutic categories revealed contrasting contraindication profiles between the pregnancy and preconception periods. Antihypertensives (code: 214), hyperlipidemia agents (code: 218), antidiabetic drugs (code: 396), and synthetic antibacterials (code: 624) displayed high contraindication rates for pregnant women (75.0, 59.1, 73.7, and 84.6%, respectively). However, these medications had no contraindications for women planning pregnancy, highlighting the temporal specificity of regulatory classifications in reproductive healthcare. This finding indicates that regulatory restrictions for these therapeutic categories are significantly limited for women planning pregnancy compared to the gestational phase. In contrast, other agents affecting metabolism (code: 399) and miscellaneous antineoplastics (code: 429) showed high contraindication rates for both pregnant women and those planning pregnancy (Figure 3c,d). This pattern was particularly evident for immunomodulators and antineoplastic agents, reflecting their distinct potential risks across both reproductive stages.

## 4. Discussion

This study represents a comprehensive analysis of prescription patterns for medications with regulatory contraindications during pregnancy and preconception periods using Japan’s national claims database. The analysis revealed a significant discrepancy between pregnancy and preconception contraindications, with 18.9% of medications contraindicated during pregnancy compared to only 5.3% of medications contraindicated during the preconception period. The findings indicate that commonly prescribed medications, such as loxoprofen sodium hydrate, sodium valproate, and metformin hydrochloride, continue to be widely prescribed to women of reproductive age (15–49 years) despite their contraindications. These results highlight the high baseline utilization of these medications in this demographic. As the majority of women in this age group are not pregnant or attempting conception, these dispensing volumes largely reflect appropriate clinical care for non-pregnant patients rather than prescribing errors. However, given the possibility of unplanned pregnancies, they underscore the importance of routine confirmation of pregnancy status during dispensing.

### 4.1. Disparities in Regulatory Classifications

The analysis demonstrated notable disparities in medication contraindication patterns between pregnant women and those planning pregnancy. As expected biologically and regulatorily, the discrepancy (18.9 vs. 5.3%) reflects differing regulatory considerations: pregnancy contraindications account for fetal effects throughout all developmental stages, whereas preconception contraindications primarily focus on risks during organogenesis. Drug exposure during early pregnancy can exert an “all or none effect” on embryonic survival [2]. However, structural congenital abnormalities are linked to drugs capable of crossing the placental barrier during organogenesis [14]. The finding that 77.3% of medications contraindicated during the preconception period are also restricted during pregnancy suggests that while preconception risks are more targeted, they largely persist into the gestational phase. While this comparison provides a descriptive overview of the current regulatory landscape in Japan, it does not directly inform actual prescribing risks or the effectiveness of preconception care, given the lack of patient-level pregnancy intention data.

### 4.2. Therapeutic Category-Specific Considerations

#### 4.2.1. Antiseizure Medications

Although antiepileptics (code: 113) exhibited a relatively low rate of pregnancy contraindications (5.9%), sodium valproate—the only antiseizure medication contraindicated during pregnancy—had a high prescription volume among women of reproductive age. Valproic acid poses a significant risk of major congenital malformations and neurodevelopmental disorders compared to other antiseizure medications [6]. The administration of valproate to women of childbearing potential should be restricted to cases where seizures are difficult to control with alternative medications. However, it must be acknowledged that for certain patients, such as those with refractory epilepsy, valproate remains clinically necessary. In such cases, the presence of these prescriptions does not inherently indicate inappropriate use; rather, it reflects complex clinical decision-making and the necessity of strict risk-management programs. Consistent with our findings of high valproate prescription volumes, a recent Japanese birth cohort study reported that some women continued to use valproate before and during early pregnancy, highlighting the real-world challenges of managing these medications [4]. Levetiracetam should be considered as a first-line treatment for conditions such as juvenile myoclonic epilepsy, given its safer profile regarding teratogenicity [15,16]. Furthermore, pharmacists can collaborate with prescribing physicians to provide supplementary education and support for women regarding the risks associated with high-dose monotherapy or polytherapy regimens [17].

#### 4.2.2. Nonsteroidal Anti-Inflammatory Drugs

Loxoprofen sodium hydrate was identified as the most frequently prescribed pregnancy-contraindicated medication, accounting for 15.7% of all pregnancy-contraindicated prescription volumes. However, it is important to note that the regulatory pregnancy-contraindication for nonsteroidal anti-inflammatory drugs (NSAIDs) in Japan typically applies only to the late stages of pregnancy (the third trimester) due to well-documented risks of ductus arteriosus constriction and oligohydramnios [18]. As our dataset lacks information on gestational timing, all NSAID prescriptions were broadly classified as potential exposures. This limitation inherently overestimates the true volume of clinically meaningful risks, as NSAID use in early pregnancy is not explicitly contraindicated. Emerging evidence suggests that these risks may extend into mid-pregnancy, highlighting the need for careful monitoring [19,20]. Given that NSAIDs are widely available as over-the-counter drugs, the potential exposure in this population may be substantially underestimated. Pharmacists can collaborate with physicians by suggesting safer alternatives—such as acetaminophen—for women who may become pregnant.

#### 4.2.3. Antidiabetics

Metformin hydrochloride accounted for 5.5% of pregnancy-contraindicated prescriptions in this study. Although listed as pregnancy-contraindicated or restricted in Japanese package inserts, metformin is considered a first-line treatment for gestational diabetes in many international guidelines due to its established safety profile. Large-scale cohort studies from Nordic countries and the United States have supported its safety regarding congenital malformations [21,22,23]. Therefore, the high prescription volume of metformin observed in our study likely reflects appropriate, evidence-based clinical practice rather than unsafe prescribing. This highlights the critical gap between regulatory package inserts and current clinical evidence in Japan. Additionally, although glucagon-like peptide-1 receptor agonists are increasingly used by young women for weight management, their safety in early pregnancy remains less well established than that of metformin, necessitating cautious management [24,25].

#### 4.2.4. Antihypertensives

Renin–angiotensin–aldosterone system inhibitors—such as olmesartan and telmisartan—were frequently prescribed to women of reproductive age. These drugs are associated with fetal renal toxicity, oligohydramnios, and potential teratogenicity in early pregnancy [26,27]. For women planning pregnancy, switching to pharmacologically safer alternatives, such as methyldopa, labetalol, or nifedipine, should be considered before conception [28,29]. However, for patients with specific conditions, such as chronic kidney disease, individualized assessment is necessary, as abrupt discontinuation may pose greater maternal risks [30].

#### 4.2.5. Reproductive Health and Obstetric Medications

It is conceptually crucial to distinguish medications used for active reproductive management or obstetric care from those representing unintended exposure risks. For example, mixed hormone preparations (code 248), such as drospirenone/ethinylestradiol, ranked highly among pregnancy-contraindicated prescriptions. However, because these agents are predominantly used as combined oral contraceptives to actively prevent pregnancy, the risk of fetal exposure is near zero. Their use reflects appropriate reproductive health management and indicates the absence of pregnancy risk.

Similarly, obstetric categories such as oxytocics (code 253) and specific uterine relaxants (e.g., ritodrine hydrochloride) are concentrated in the reproductive-age demographic because they represent intentional pregnancy-related care. Indeed, a recent Japanese birth cohort study demonstrated that ritodrine hydrochloride is widely used as a uterine relaxant in Japan, becoming the most frequently prescribed medication (10.5%) after 12 weeks of gestation [4]. Including these reproductive and obstetric medications in our aggregated analysis inherently inflates the overall estimate of unintended exposure risks. Therefore, the high prescription volumes in these specific categories should be interpreted as appropriate, indication-specific clinical care rather than safety concerns or gaps in preconception care.

### 4.3. Regulatory and Pharmacological Discrepancies

It is crucial to recognize that regulatory package inserts may not always reflect the most current clinical evidence regarding drug safety during pregnancy. For instance, domperidone was identified as a frequently prescribed pregnancy-contraindicated medication in this study (Table 2). While listed as pregnancy-contraindicated in Japanese package inserts, recent observational studies have suggested no significant increase in major congenital malformations following first-trimester exposure [31,32]. The Guidelines for Obstetrical Practice in Japan also note that some contraindicated drugs lack clinically significant fetal effects when used in early pregnancy. This region-specific lag between accumulating safety data and regulatory updates creates a dilemma for local clinicians. Consequently, high prescription volumes for certain pregnancy-contraindicated medications do not necessarily imply inappropriate prescribing; rather, they may represent clinical practice prioritizing current evidence over outdated labels. This highlights the need for pharmacists to provide counseling based on the latest evidence rather than relying solely on package inserts. Furthermore, pharmacological properties should be considered when evaluating potential exposure risks. Our analysis revealed that rosuvastatin calcium was the most frequently prescribed statin among women of reproductive age (Table 2), despite all statins being contraindicated for pregnant women in Japan. Pharmacologically, hydrophilic statins, such as pravastatin, have negligible placental transfer and minimal fetal exposure compared with lipophilic statins. Given that pravastatin has been investigated for preventing preeclampsia due to its favorable safety profile [33], the predominance of rosuvastatin prescriptions suggests an opportunity for pharmacists to recommend switching to pharmacologically safer alternatives for women planning pregnancy, even within the same pregnancy-contraindicated therapeutic class.

### 4.4. Limitations

This study has some limitations. First, the NDB Open Data provides only aggregated prescription volumes and lacks individual patient identifiers, preventing linkage to pregnancy outcomes or determination of whether women were pregnant or planning pregnancy. Therefore, our results reflect potential exposure in women of reproductive age rather than confirmed exposure during pregnancy. Second, the outcome metric relies on the sum of drug price calculation units, which mixes various units such as tablets, capsules, grams, and milliliters. Without normalization to defined daily doses or the number of patients, which is mathematically impossible due to the aggregated nature of the NDB Open Data, direct comparisons across different medications and therapeutic categories are inherently flawed. Consequently, medications prescribed in powders or liquids (measured in grams or milliliters) may appear artificially inflated in total volume compared to those prescribed in tablets. Therefore, the presented rankings of ‘high prescription volume’ should be interpreted with caution, as they primarily reflect the scale of dispensed physical units rather than true exposure prevalence or actual patient counts. Third, medications were classified using Japanese standard therapeutic category codes rather than the Anatomical Therapeutic Chemical classification system. While this limits direct international comparisons, these codes are standard for insurance claims and regulatory purposes in Japan, making them most relevant for domestic policy and package insert revisions. Fourth, the database lacks diagnostic information. For medications with multiple indications (e.g., sodium valproate for epilepsy vs. migraine), the prescribed indication could not be confirmed. As we conservatively classified such drugs as contraindicated if any approved indication carried a pregnancy contraindication, this approach inherently introduces a misclassification bias. Consequently, our findings likely overestimate the true volume of contraindicated medications intended for restricted indications. Due to the aggregated nature of the data, an indication-specific sensitivity analysis was not feasible. However, this conservative approach was adopted to capture the maximum scope of potential exposure. Furthermore, our classification system grouped all pregnancy-contraindicated medications into a single category, regardless of trimester-specific restrictions. For example, NSAIDs are contraindicated only in late pregnancy; however, due to the lack of gestational age data, we could not distinguish prescriptions by trimester. This conservative grouping further contributes to the overestimation of clinically meaningful exposure risks. Finally, the database reports only the top-ranking items in each therapeutic category (e.g., top 100 or 500 items), and over-the-counter medications are not captured, potentially underestimating total drug exposure. Moreover, as these findings heavily rely on the unique regulatory classifications and healthcare system of Japan, they are highly region-specific and should not be directly extrapolated to other countries with differing medical guidelines and regulatory frameworks.

### 4.5. Generalizability

The generalizability of these findings warrants careful consideration. This study utilized Japan’s NDB Open Data, which covers nearly the entire Japanese population (98.8% of medical claims and 99.8% of dispensing claims), providing robust national representativeness within Japan. However, the findings are inherently specific to the Japanese healthcare system, as medication regulatory classifications, prescribing practices, and the scope of pharmacist interventions vary substantially across countries. For example, metformin is contraindicated during pregnancy in Japanese package inserts but is recommended as a first-line treatment for gestational diabetes in many international guidelines. Therefore, direct extrapolation of these results to other countries with differing regulatory frameworks and clinical guidelines should be avoided.

## 5. Conclusions

This study highlights the expected regulatory differences in medication classifications between pregnancy and preconception periods and identifies a high volume of potential exposure to regulatory pregnancy-contraindicated medications among women of reproductive age in Japan. Specifically, high prescription volumes of loxoprofen, valproate, and metformin underscore the need for rigorous medication reviews. Recognizing that most prescriptions in this demographic are appropriately intended for non-pregnant women, pharmacists can collaborate with prescribing physicians by routinely confirming pregnancy status to identify potential inadvertent exposures. Future efforts should aim to align package insert warnings with current clinical evidence to support more appropriate clinical decision-making.

## Figures and Tables

**Table 1:** Prescription volumes of pregnancy-contraindicated medications prescribed to women of reproductive age and comparisons with the general population, according to therapeutic category code.

Therapeutic Category Code	Therapeutic Category	Outpatient Prescription Volume (Prescription Units)		Proportions (%)	
		Women Aged 15–49 Years (A)	All Sex and Age Groups (B)	(A)/(B)	Share of all Pregnancy Contraindications (Women Aged 15–49 Years)
114	Antipyretics and analgesics, anti-inflammatory agents	279,345,055	2,225,914,550	12.5	20.4
249	Miscellaneous hormones	135,204,121	362,522,156	37.3	9.9
396	Antidiabetic agents	135,173,437	4,793,906,308	2.8	9.9
113	Antiepileptics	127,995,210	632,682,489	20.2	9.4
248	Mixed hormone preparations	126,686,442	129,869,888	97.5	9.3
218	Hyperlipidemia agents	103,794,040	5,284,047,324	2.0	7.6
214	Antihypertensives	88,075,841	5,182,404,435	1.7	6.4
117	Psychotropics	63,995,507	304,012,110	21.1	4.7
399	Other agents affecting metabolism	41,297,407	570,629,598	7.2	3.0
239	Other agents affecting the digestive system	37,796,119	215,162,212	17.6	2.8
219	Other agents affecting the cardiovascular system	37,770,549	384,353,588	9.8	2.8
247	Estrogen and progesterone preparations	26,511,530	54,684,171	48.5	1.9
429	Miscellaneous antineoplastics	25,386,284	179,094,506	14.2	1.9
339	Other agents affecting blood and body fluids	22,741,741	2,295,307,415	1.0	1.7
624	Synthetic antibacterials	19,174,121	90,760,945	21.1	1.4
449	Miscellaneous allergic agents	15,365,012	60,718,388	25.3	1.1
217	Vasodilators	10,574,149	612,771,781	1.7	0.8
311	Vitamins A and D preparations	10,239,822	739,510,376	1.4	0.7
259	Other agents affecting the urogenital and anal organ system	10,080,112	218,691,006	4.6	0.7
333	Anticoagulants	9,179,644	498,378,847	1.8	0.7
629	Miscellaneous chemotherapeutics	8,656,052	104,467,854	8.3	0.6
235	Purgatives, clysters	6,974,951	207,555,525	3.4	0.5
212	Antiarrhythmic agents	4,384,851	167,654,208	2.6	0.3
116	Antiparkinsonian agents	3,663,739	154,564,375	2.4	0.3
394	Gout preparations	3,538,370	110,147,897	3.2	0.3
325	Protein and amino acid preparations	3,142,634	86,650,596	3.6	0.2
422	Antimetabolites	3,131,907	86,880,502	3.6	0.2
253	Oxytocics	1,226,770	1,233,631	99.4	0.1
213	Diuretics	1,226,600	50,485,932	2.4	0.1
317	Multivitamin preparations	781,519	7,654,252	10.2	0.1
625	Antivirals	774,212	16,702,737	4.6	0.1
393	Habitual intoxication agents	728,983	7,926,371	9.2	0.1
119	Other agents affecting the central nervous system	394,582	3,381,125	11.7	0.0
392	Antidotes	286,368	4,095,004	7.0	0.0
617	Acting mainly on mold	262,859	6,146,991	4.3	0.0
123	Autonomic nervous system agents	183,968	11,356,513	1.6	0.0
232	Peptic ulcer agents	153,521	3,214,412	4.8	0.0
421	Alkylating agents	63,647	571,092	11.1	0.0
244	Anabolic steroid preparations	56,005	7,206,908	0.8	0.0
236	Cholagogues	20,302	86,598	23.4	0.0
622	Tuberculosis preparations	17,244	39,700	43.4	0.0
442	Stimulation therapy agents	6762	421,811	1.6	0.0
641	Antiprotozoans	4291	12,621	34.0	0.0
642	Anthelmintics	3856	66,209	5.8	0.0
424	Plant extract preparations	3259	131,641	2.5	0.0
619	Miscellaneous antibiotics	1211	21,665	5.6	0.0
621	Sulfonamides	<1000	<1000	N/A	N/A

Note: The outpatient prescription volume represents the sum of drug price calculation units (e.g., tablets, capsules, grams, milliliters) and does not represent the number of patients or prescriptions. Data represent potential exposure in women of reproductive age (15–49 years), not confirmed exposure in pregnant women. N/A = not applicable; the exact numerator is unknown (reported only as <1000), so percentages cannot be derived.

**Table 2:** Top 50 pregnancy-contraindicated medications prescribed to women of reproductive age and comparisons with the general population.

Therapeutic Category Code	Active Pharmaceutical Ingredients of Oral Medications	Outpatient Prescription Volume (Prescription Units)		Proportions (%)		
		Women Aged 15–49 Years (A)	All Sex and Age Groups (B)	(A)/(B)	(A)/All Pregnancy-Contraindicated Agents for Women Aged 15–49 Years	(A)/Agents Within the Therapeutic Category Code for Women Aged 15–49 Years
114	Loxoprofen sodium hydrate	214,857,939	1,334,696,921	16.1	15.7	42.4
113	Sodium valproate	127,995,210	632,682,489	20.2	9.4	36.3
249	Dienogest	120,798,129	135,590,267	89.1	8.8	83.4
396	Metformin hydrochloride	74,859,586	2,058,251,220	3.6	5.5	48.0
248	Drospirenone/ethinylestradiol betadex combination	62,798,390	63,410,556	99.0	4.6	49.6
248	Norethisterone/ethinylestradiol combination	52,960,625	53,584,017	98.8	3.9	41.8
117	Lithium carbonate	43,122,063	164,422,985	26.2	3.2	5.1
218	Rosuvastatin calcium	37,244,366	1,781,044,912	2.1	2.7	30.9
239	Domperidone	37,156,699	213,084,616	17.4	2.7	13.4
219	Lomerizine hydrochloride	33,273,563	70,419,881	47.3	2.4	35.4
114	Celecoxib	28,992,890	625,919,850	4.6	2.1	5.7
429	Tamoxifen citrate	20,221,476	48,456,887	41.7	1.5	72.8
218	Atorvastatin calcium hydrate	17,227,943	1,088,152,468	1.6	1.3	14.3
399	Mycophenolate mofetil	16,630,384	62,885,422	26.4	1.2	8.2
218	Pitavastatin calcium hydrate	15,767,007	809,877,133	1.9	1.2	13.1
114	Diclofenac sodium	14,679,927	111,616,552	13.2	1.1	2.9
218	Pemafibrate	14,336,561	433,614,145	3.3	1.0	11.9
214	Olmesartan medoxomil	12,860,660	681,538,983	1.9	0.9	11.3
449	Tranilast	12,762,248	41,294,664	30.9	0.9	1.3
247	Conjugated estrogens	12,232,477	18,648,681	65.6	0.9	25.0
214	Telmisartan	10,932,196	572,769,938	1.9	0.8	9.6
114	Ibuprofen	9,929,399	34,868,563	28.5	0.7	2.0
214	Azilsartan	9,762,405	474,538,470	2.1	0.7	8.6
399	Methotrexate (indication: rheumatism)	9,273,876	83,877,326	11.1	0.7	4.6
214	Candesartan cilexetil	8,916,317	545,514,534	1.6	0.7	7.8
333	Warfarin potassium	8,536,447	353,769,568	2.4	0.6	71.8
259	Ritodrine hydrochloride	8,461,537	8,466,612	99.9	0.6	39.4
311	Eldecalcitol	8,062,335	728,134,293	1.1	0.6	31.6
339	Limaprost alfadex	7,792,499	913,280,499	0.9	0.6	23.0
396	Dapagliflozin propylene glycolate hydrate	7,505,221	233,766,551	3.2	0.5	4.8
117	Guanfacine hydrochloride	7,334,690	44,204,670	16.6	0.5	0.9
248	Levonorgestrel/ethinylestradiol combination	7,266,171	7,376,736	98.5	0.5	5.7
247	Medroxyprogesterone acetate	7,237,990	14,149,551	51.2	0.5	14.8
339	Aspirin	7,044,988	751,539,878	0.9	0.5	20.8
117	Haloperidol	7,043,845	49,743,587	14.2	0.5	0.8
235	Lubiprostone	6,974,951	207,555,525	3.4	0.5	9.7
396	Vildagliptin/metformin hydrochloride combination	6,852,092	341,343,451	2.0	0.5	4.4
624	Levofloxacin hydrate	6,459,596	36,265,133	17.8	0.5	33.7
249	Relugolix	6,217,903	8,205,128	75.8	0.5	4.3
396	Empagliflozin	6,202,787	184,270,890	3.4	0.5	4.0
218	Pravastatin sodium	5,837,825	455,480,997	1.3	0.4	4.8
399	Iguratimod	5,827,885	64,452,007	9.0	0.4	2.9
218	Bezafibrate	5,704,794	245,654,297	2.3	0.4	4.7
249	Semaglutide	5,182,797	62,285,632	8.3	0.4	3.6
247	Estradiol	5,038,172	12,743,564	39.5	0.4	10.3
396	Glimepiride	4,955,282	393,242,984	1.3	0.4	3.2
214	Telmisartan/amlodipine besilate combination	4,903,671	274,569,366	1.8	0.4	4.3
629	Trimethoprim/sulfamethoxazole combination	4,891,578	60,062,091	8.1	0.4	48.5
214	Irbesartan/amlodipine besilate combination	4,860,598	274,636,630	1.8	0.4	4.3
214	Losartan potassium	4,708,192	189,315,005	2.5	0.3	4.1

Note: Active pharmaceutical ingredients of oral medications were classified as pregnancy-contraindicated based on the Pregnant Women section of the Japanese package inserts. Prescription volume indicates the total count of calculation units (e.g., tablets, grams). (A)/(B): Proportion of prescriptions for women aged 15–49 years relative to the total prescriptions for all sexes and age groups. (A)/all pregnancy-contraindicated agents for women aged 15–49 years: Proportion of the specific agent’s prescription volume relative to the total prescription volume of all pregnancy-contraindicated medications prescribed to women aged 15–49 years. (A)/agents within the therapeutic category code for women aged 15–49 years: Proportion of the specific agent’s prescription volume relative to the total prescription volume of all medications within its respective therapeutic category prescribed to women aged 15–49 years.

## Data Availability

The raw data supporting the conclusions of this article will be made available by the authors on request.

## Supplementary Materials

The following supporting information can be downloaded at: https://www.mdpi.com/article/10.3390/pharmacy14020051/s1, Table S1: STROBE checklist for cross-sectional studies; Table S2: List of all 1425 active pharmaceutical ingredients of oral medications with their regulatory classifications for pregnancy and preconception.

## Abbreviations

ACE	Angiotensin-Converting Enzyme
ARB	Angiotensin Receptor Blocker
FDA	Food and Drug Administration
FY	Fiscal Year
STROBE	Strengthening the Reporting of Observational Studies in Epidemiology
NDB	National Database of Health Insurance Claims and Specific Health Checkups of Japan
NSAIDs	Non-Steroidal Anti-Inflammatory Drugs
PCC	Preconception Care
PMDA	Pharmaceuticals and Medical Devices Agency
WHO	World Health Organization

## References

[1] van Gelder M.M.H.J., van Rooij I.A.L.M., Miller R.K., Zielhuis G.A., de Jong-van den Berg L.T.W., Roeleveld N. (2010). Teratogenic Mechanisms of Medical Drugs. Hum. Reprod. Update.

[2] Adam M.P. (2012). The All-or-None Phenomenon Revisited. Birth Defects Res. Part A Clin. Mol. Teratol..

[3] Nishigori H., Obara T., Nishigori T., Metoki H., Ishikuro M., Mizuno S., Sakurai K., Tatsuta N., Nishijima I., Fujiwara I. (2017). Drug Use before and during Pregnancy in Japan: The Japan Environment and Children’s Study. Pharmacy.

[4] Noda A., Obara T., Shirota M., Ueno F., Matsuzaki F., Hatanaka R., Obara R., Morishita K., Shinoda G., Orui M. (2024). Medication Use before and during Pregnancy in Japan: The Tohoku Medical Megabank Project Birth and Three-Generation Cohort Study. Eur. J. Clin. Pharmacol..

[5] WHO (2013). World Health Organization Meeting Report. Meeting to Develop a Global Consensus on Preconception Care to Reduce Maternal and Childhood Mortality and Morbidity.

[6] Battino D., Tomson T., Bonizzoni E., Craig J., Perucca E., Sabers A., Thomas S., Alvestad S., Perucca P., Vajda F. (2024). Risk of Major Congenital Malformations and Exposure to Antiseizure Medication Monotherapy. JAMA Neurol..

[7] Ministry of Health, Labour and Welfare NDB Open Data Japan. https://www.mhlw.go.jp/stf/seisakunitsuite/bunya/0000177182.html.

[8] von Elm E., Altman D.G., Egger M., Pocock S.J., Gøtzsche P.C., Vandenbroucke J.P. (2007). STROBE Initiative Strengthening the Reporting of Observational Studies in Epidemiology (STROBE) Statement: Guidelines for Reporting Observational Studies. BMJ.

[9] von Elm E., Altman D.G., Egger M., Pocock S.J., Gøtzsche P.C., Vandenbroucke J.P. (2007). The Strengthening the Reporting of Observational Studies in Epidemiology (STROBE) Statement: Guidelines for Reporting Observational Studies. Ann. Intern. Med..

[10] Ministry of Health, Labour and Welfare Changes in Electric Claims Data at Medical Institution Based on Numbers of Claims Data. https://www.ssk.or.jp/tokeijoho/tokeijoho_rezept/tokeijoho_rezept_r04.files/seikyu_0503.pdf.

[11] Ura H., Senoo M., Kubota K., Sadamoto K. (2024). The Current Status of Utilizing a Medication Record Handbook for Evaluating Shared Medication History: A Retrospective Study Using the Japanese National Claims Database. Cureus.

[12] Ura H., Matsuoka N., Kubota K., Sadamoto K. (2024). Trends in Prescription of Anti-Seizure Medications in Japan between 2018 and 2021: A Retrospective Study Using the National Database Open Data Japan. Epilepsy Behav..

[13] Pharmaceuticals and Medical Devices Agency Search System for Prescription-Drugs. https://www.pmda.go.jp/PmdaSearch/iyakuSearch.

[14] Pacifici G.M., Nottoli R. (1995). Placental Transfer of Drugs Administered to the Mother. Clin. Pharmacokinet..

[15] Janz D. (1985). Epilepsy with Impulsive Petit Mal (Juvenile Myoclonic Epilepsy). Acta Neurol. Scand..

[16] Ascoli M., Mastroianni G., Gasparini S., Striano P., Cianci V., Neri S., Bova V., Mammì A., Gambardella A., Labate A. (2021). Diagnostic and Therapeutic Approach to Drug-Resistant Juvenile Myoclonic Epilepsy. Expert Rev. Neurother..

[17] Veroniki A.A., Cogo E., Rios P., Straus S.E., Finkelstein Y., Kealey R., Reynen E., Soobiah C., Thavorn K., Hutton B. (2017). Comparative Safety of Anti-Epileptic Drugs during Pregnancy: A Systematic Review and Network Meta-Analysis of Congenital Malformations and Prenatal Outcomes. BMC Med..

[18] Dathe K., Hultzsch S., Pritchard L.W., Schaefer C. (2019). Risk Estimation of Fetal Adverse Effects after Short-Term Second Trimester Exposure to Non-Steroidal Anti-Inflammatory Drugs: A Literature Review. Eur. J. Clin. Pharmacol..

[19] Dathe K., Frank J., Padberg S., Hultzsch S., Beck E., Schaefer C. (2022). Fetal Adverse Effects Following NSAID or Metamizole Exposure in the 2nd and 3rd Trimester: An Evaluation of the German Embryotox Cohort. BMC Pregnancy Childbirth.

[20] Kolding L., Eken H., Uldbjerg N. (2020). Drug Exposure during Pregnancy and Fetal Cardiac Function—A Systematic Review. J. Perinat. Med..

[21] Cassina M., Donà M., Di Gianantonio E., Litta P., Clementi M. (2014). First-Trimester Exposure to Metformin and Risk of Birth Defects: A Systematic Review and Meta-Analysis. Hum. Reprod. Update.

[22] Kjerpeseth L.J., Cesta C.E., Furu K., Engeland A., Gissler M., Gulseth H.L., Karlstad Ø., Leinonen M.K., Pazzagli L., Zoega H. (2023). Metformin versus Insulin and Risk of Major Congenital Malformations in Pregnancies with Type 2 Diabetes: A Nordic Register-Based Cohort Study. Diabetes Care.

[23] Chiu Y.-H., Huybrechts K.F., Patorno E., Yland J.J., Cesta C.E., Bateman B.T., Seely E.W., Hernán M.A., Hernández-Díaz S. (2024). Metformin Use in the First Trimester of Pregnancy and Risk for Nonlive Birth and Congenital Malformations: Emulating a Target Trial Using Real-World Data. Ann. Intern. Med..

[24] Cesta C.E., Rotem R., Bateman B.T., Chodick G., Cohen J.M., Furu K., Gissler M., Huybrechts K.F., Kjerpeseth L.J., Leinonen M.K. (2024). Safety of GLP-1 Receptor Agonists and Other Second-Line Antidiabetics in Early Pregnancy. JAMA Intern. Med..

[25] Han S.H., Ockerman K., Furnas H., Mars P., Klenke A., Ching J., Momeni A., Sorice-Virk S. (2024). Practice Patterns and Perspectives of the Off-Label Use of GLP-1 Agonists for Cosmetic Weight Loss. Aesthet. Surg. J..

[26] Korkes H.A., de Oliveira L.G., Berlinck L., Goes F.S., Borges A.F.A., Kirsztajn G.M., Sass N. (2014). Human Fetal Malformations Associated with the Use of an Angiotensin II Receptor Antagonist: Case Report. J. Bras. Nefrol..

[27] Fu J., Tomlinson G., Feig D.S. (2021). Increased Risk of Major Congenital Malformations in Early Pregnancy Use of Angiotensin-Converting-Enzyme Inhibitors and Angiotensin-Receptor-Blockers: A Meta-Analysis. Diabetes/Metab. Res. Rev..

[28] Yin J., Mei Z., Shi S., Du P., Qin S. (2022). Nifedipine or Amlodipine? The Choice for Hypertension during Pregnancy: A Systematic Review and Meta-Analysis. Arch. Gynecol. Obstet..

[29] Nassar A.H., Abu-Musa A.A., Awwad J., Khalil A., Tabbara J., Usta I.M. (2009). Two Dose Regimens of Nifedipine for Management of Preterm Labor: A Randomized Controlled Trial. Am. J. Perinatol..

[30] Pucci M., Sarween N., Knox E., Lipkin G., Martin U. (2015). Angiotensin-Converting Enzyme Inhibitors and Angiotensin Receptor Blockers in Women of Childbearing Age: Risks versus Benefits. Expert Rev. Clin. Pharmacol..

[31] Hishinuma K., Yamane R., Yokoo I., Arimoto T., Takahashi K., Goto M., Saito Y., Nakajima K., Murashima A., Hayashi M. (2021). Pregnancy Outcome after First Trimester Exposure to Domperidone—An Observational Cohort Study. J. Obstet. Gynaecol. Res..

[32] Ishikawa T., Obara T., Akazawa M., Noda A., Oyanagi G., Morishita K., Miyakoda K., Nishigori H., Kawame H., Yaegashi N. (2022). Risk of Major Congenital Malformations Associated with First-Trimester Exposure to Propulsives: A Health Administrative Database Study in Japan. Pharmacoepidemiol. Drug Saf..

[33] Costantine M.M., Cleary K., Hebert M.F., Ahmed M.S., Brown L.M., Ren Z., Easterling T.R., Haas D.M., Haneline L.S., Caritis S.N. (2016). Safety and Pharmacokinetics of Pravastatin Used for the Prevention of Preeclampsia in High-Risk Pregnant Women: A Pilot Randomized Controlled Trial. Am. J. Obstet. Gynecol..

