# Supplementary material for: Assessment of Potential Exposure to Pregnancy-Contraindicated Medications Among Women of Reproductive Age in Japan: A Retrospective Database Study
Source: Pharmacy (Basel). 2026 Mar 20;14(2):51. doi: 10.3390/pharmacy14020051 (PMC13010656; doi:10.3390/pharmacy14020051)
Supplement: Supplementary file 1 [file pharmacy-14-00051-s001.zip › pharmacy-4170184-supplementary.pdf]

Table S1. STROBE checklist for cross-sectional studies

	Item Description	Location (or reason for not reporting)
**Title and abstract**		
1a. Indicate the study's design	Indicate the study's design with a commonly used term in the title or the abstract.	Title: "retrospective database study"; Abstract: "retrospective cross-sectional study"
1b. Abstract	Provide in the abstract an informative and balanced summary of what was done and what was found.	Abstract, p.1
**Introduction**		
2. Background / rationale	Explain the scientific background and rationale for the investigation being reported.	Introduction, Section 1, pp.1–2
3. Objectives	State specific objectives, including any prespecified hypotheses.	Introduction, Section 1, p.2: "the primary objective of this study was to assess the potential exposure of women of reproductive age to pregnancy-contraindicated medications in Japan"
**Methods**		
4. Study design	Present key elements of study design early in the paper.	Methods, Section 2.1, p.2: "retrospective cross-sectional descriptive study"
5. Setting	Describe the setting, locations, and relevant dates, including periods of recruitment, exposure, follow-up, and data collection.	Methods, Section 2.1, p.2: NDB Open Data Japan, FY 2022 (April 1, 2022 to March 31, 2023)
6a. Eligibility criteria	**Cohort study:** Give the eligibility criteria, and the sources and methods of selection of participants. Describe methods of follow-up. **Case-control study:** Give the eligibility criteria, and the sources and methods of case ascertainment and control selection. Give the rationale for the choice of cases and controls. **Cross-sectional study:** Give the eligibility criteria, and the sources and methods of selection of participants.	Methods, Section 2.2, pp.2–3: women aged 15–49 years; oral medications from outpatient prescriptions
6b. Matching criteria	**Cohort study:** For matched studies, give matching criteria and number of exposed and unexposed. **Case-control study:** For matched studies, give matching criteria and the number of controls per case.	Not applicable; this is a descriptive cross-sectional study without matching
7. Variables	Clearly define all outcomes, exposures, predictors, potential confounders, and effect modifiers. Give diagnostic criteria, if applicable.	Methods, Section 2.3, p.3: three classification categories (contraindicated, benefit outweighs risk, no cautionary statement); Section 2.4, p.3:

		prescription volume as primary outcome
8. Data sources / measurement	For each variable of interest give sources of data and details of methods of assessment (measurement). Describe comparability of assessment methods if there is more than one group.	Methods, Section 2.1, p.2: NDB Open Data Japan; Section 2.3, p.3: PMDA package insert search system; two independent reviewers
9. Bias	Describe any efforts to address potential sources of bias.	Methods, Section 2.3, p.3: two independent reviewers with discrepancy resolution through discussion; conservative classification approach; Limitations, Section 4.4, pp.14–15
10. Study size	Explain how the study size was arrived at.	Results, Section 3.1, p.4: all available data from the NDB Open Data were included; 8,387 oral medications consolidated into 1,425 active ingredients
11. Quantitative variables	Explain how quantitative variables were handled in the analyses. If applicable, describe which groupings were chosen, and why.	Methods, Section 2.4, p.3: prescription volume calculated as sum of drug price calculation units; protein and amino acid preparations measured in packaging units
12a. Statistical methods	Describe all statistical methods, including those used to control for confounding.	Methods, Section 2.4, p.3: descriptive statistics; Python (v. 3.10.12), NumPy (v. 1.26.4), Pandas (v. 2.2.2)
12b. Statistical methods – subgroups and interactions	Describe any methods used to examine subgroups and interactions.	Methods, Section 2.4, p.3: analysis stratified by therapeutic category codes and age group (15–49 years vs. all sex and age groups)
12c. Statistical methods – missing data	Explain how missing data were addressed.	Results, Section 3.1, p.4: aggregated data without individual-level records; missing data assessment not applicable; database coverage 98.8–99.8%
12di. Statistical methods – loss to follow-up	**Cohort study:** If applicable, describe how loss to follow-up was addressed.	Not applicable; cross-sectional study
12dii. Statistical methods – matching cases and controls	**Case-control study:** If applicable, explain how matching of cases and controls was addressed.	Not applicable; cross-sectional study

12diii. Statistical methods – sampling strategy	**Cross-sectional study:** If applicable, describe analytical methods taking account of sampling strategy.	Not applicable; the NDB Open Data covers nearly the entire Japanese population (98.8% of medical claims and 99.8% of dispensing claims)
12e. Statistical methods – sensitivity analyses	Describe any sensitivity analyses.	Limitations, Section 4.4, p.14: acknowledged that indication-specific sensitivity analysis was not feasible due to aggregated nature of data
**Results**		
13a. Participant numbers	Report the numbers of individuals at each stage of the study—e.g., numbers potentially eligible, examined for eligibility, confirmed eligible, included in the study, completing follow-up, and analysed; Consider use of a flow diagram.	Results, Section 3.1, p.4; Figure 1: 8,387 oral medications → 1,425 active pharmaceutical ingredients → classified into three categories
13b. Participants – non-participation	Give reasons for non-participation at each stage.	Not applicable; aggregated national claims data with no individual-level selection
13c. Participants – flow diagram	Consider use of a flow diagram.	Figure 1, p.4
14a. Descriptive data – participant characteristics	Give characteristics of study participants (e.g., demographic, clinical, social) and information on exposures and potential confounders. Present the information in a table.	Results, Sections 3.1–3.4; Tables 1–2; Figures 2–3
14b. Descriptive data – missing data	Indicate the number of participants with missing data for each variable of interest.	Results, Section 3.1, p.4: individual-level missing data not applicable; database coverage 98.8% (medical claims) and 99.8% (dispensing claims)
14c. Descriptive data – follow-up time	**Cohort study:** Summarise follow-up time—e.g., average and total amount.	Not applicable; cross-sectional study
15. Outcome data	**Cohort study:** Report numbers of outcome events or summary measures over time. **Case-control study:** Report numbers in each exposure category, or summary measures of exposure. **Cross-sectional study:** Report numbers of outcome events or summary measures.	Tables 1–2, pp.7–10: prescription volumes and proportions reported
16a. Main results	Give unadjusted estimates and, if applicable, confounder-adjusted estimates and their precision (e.g., 95% confidence intervals). Make clear which confounders were adjusted for and why they were included.	Tables 1–2: prescription volumes and proportions reported; no confounder-adjusted estimates (descriptive study using aggregated data)
16b. Main results – category boundaries	Report category boundaries when continuous variables were categorised.	Methods, Section 2.2, p.2: age category 15–49 years (five-year age groups); Section 2.3, p.3:

		three medication classification categories
16c. Main results – risk	If relevant, consider translating estimates of relative risk into absolute risk for a meaningful time period.	Not applicable; descriptive study without risk estimation
17. Other analyses	Report other analyses done—e.g., analyses of subgroups and interactions, and sensitivity analyses.	Results, Section 3.5, pp.10–12: Venn diagram analysis comparing pregnancy and preconception contraindications; heat map analysis across therapeutic categories
**Discussion**		
18. Key results	Summarise key results with reference to study objectives.	Discussion, Section 4, p.11
19. Limitations	Discuss limitations of the study, taking into account sources of potential bias or imprecision. Discuss both direction and magnitude of any potential bias.	Discussion, Section 4.4, p.15
20. Interpretation	Give a cautious overall interpretation considering objectives, limitations, multiplicity of analyses, results from similar studies, and other relevant evidence.	Discussion, Sections 4.1–4.3, pp.12–15
21. Generalisability	Discuss the generalisability (external validity) of the study results.	Discussion, Section 4.5, pp.15–16: national representativeness within Japan discussed; region-specific limitations acknowledged
**Other information**		
22. Funding	Give the source of funding and the role of the funders for the present study and, if applicable, for the original study on which the present article is based.	Funding, p.16: Research Grant Program for Pharmaceutical Sciences (2024) of the Kanagawa Pharmaceutical Association, grant number 24-3; the funders had no role in the study design, data collection, analysis, or manuscript preparation

Table S2. List of all 1,425 active pharmaceutical ingredients of oral medications with their regulatory classifications for pregnancy and preconception. Classifications are based on Japanese package inserts. "Use if benefit outweighs risk" indicates medications permitted only when the therapeutic benefits are judged to outweigh the potential risks.

Therapeutic category code	Active pharmaceutical ingredients of oral medications	Regulatory classification for pregnant women	Regulatory classification for women planning pregnancy	Out-of-hospital prescription volume for women aged 15-49 years (prescription-unit)
113	Sodium valproate	Pregnancy-contraindicated	Contraceptive recommendation	127995210
113	Lamotrigine	Use if benefit outweighs risk	No cautionary statement	54289579
113	Clonazepam	Use if benefit outweighs risk	No cautionary statement	46164823
113	Levetiracetam	Use if benefit outweighs risk	No cautionary statement	45772266
113	Carbamazepine	Use if benefit outweighs risk	No cautionary statement	30235930
113	Lacosamide	Use if benefit outweighs risk	No cautionary statement	12517291
113	Zonisamide (indication: epilepsy)	Use if benefit outweighs risk	No cautionary statement	7496092
113	Topiramate	Use if benefit outweighs risk	No cautionary statement	7096819
113	Clobazam	Use if benefit outweighs risk	No cautionary statement	5926058
113	Phenytoin	Use if benefit outweighs risk	No cautionary statement	5120385
113	Perampanel hydrate	Use if benefit outweighs risk	No cautionary statement	4738854
113	Gabapentin	Use if benefit outweighs risk	No cautionary statement	3025195
113	Rufinamide	Use if benefit outweighs risk	No cautionary statement	1133565
113	Hydantol F (phenytoin/phenobarbital) combination	Use if benefit outweighs risk	No cautionary statement	619297
113	Ethosuximide	Use if benefit outweighs risk	No cautionary statement	473566
113	Sultiame	Use if benefit outweighs risk	No cautionary statement	365812
113	Hydantol D (phenytoin/phenobarbital) combination	Use if benefit outweighs risk	No cautionary statement	78584
114	Loxoprofen sodium hydrate	Pregnancy-contraindicated	No cautionary statement	214857939

114	Acetaminophen	Use if benefit outweighs risk	No cautionary statement	161227270
114	Celecoxib	Pregnancy-contraindicated	No cautionary statement	28992890
114	Neurotropin	Use if benefit outweighs risk	No cautionary statement	23741293
114	Tramadol hydrochloride/Acetaminophen combination	Use if benefit outweighs risk	No cautionary statement	18451903
114	Diclofenac sodium	Pregnancy-contraindicated	No cautionary statement	14679927
114	Isopropylantipyrine/Acetaminophen/Allylisopropylacetylurea/Anhydrous Caffeine combination	Use if benefit outweighs risk	No cautionary statement	10753661
114	Ibuprofen	Pregnancy-contraindicated	No cautionary statement	9929399
114	Tramadol hydrochloride	Use if benefit outweighs risk	No cautionary statement	8597127
114	Lornoxicam	Pregnancy-contraindicated	No cautionary statement	3300631
114	Etodolac	Pregnancy-contraindicated	No cautionary statement	2759836
114	Tiaramide hydrochloride	Use if benefit outweighs risk	No cautionary statement	2650094
114	Naproxen	Pregnancy-contraindicated	No cautionary statement	2346088
114	Zaltoprofen	Use if benefit outweighs risk	No cautionary statement	2024870
114	Mefenamic acid	Pregnancy-contraindicated	No cautionary statement	1802403
114	Indometacin farnesil	Pregnancy-contraindicated	No cautionary statement	542064
114	Meloxicam	Pregnancy-contraindicated	No cautionary statement	133880
116	Biperiden hydrochloride	Use if benefit outweighs risk	No cautionary statement	36466079
116	Trihexyphenidyl hydrochloride	Use if benefit outweighs risk	No cautionary statement	9551886
116	Pramipexole hydrochloride hydrate	Pregnancy-contraindicated	No cautionary statement	2445595
116	Bromocriptine mesilate	Use if benefit outweighs risk	No cautionary statement	1598809
116	Cabergoline	Use if benefit outweighs risk	No cautionary statement	1319443
116	Levodopa/Carbidopa hydrate combination	Use if benefit outweighs risk	No cautionary statement	1212477
116	Amantadine hydrochloride	Pregnancy-contraindicated	No cautionary statement	653911
116	Levodopa/Benserazide hydrochloride combination	Use if benefit outweighs risk	No cautionary statement	375435
116	Droxidopa	Pregnancy-contraindicated	No cautionary statement	352188
116	Ropinirole hydrochloride	Pregnancy-contraindicated	No cautionary statement	110586

116	Levodopa/Carbidopa hydrate/Entacapone combination	Use if benefit outweighs risk	No cautionary statement	103402
116	Selegiline hydrochloride	Use if benefit outweighs risk	No cautionary statement	87678
116	Trihexyphenidyl hydrochloride	Use if benefit outweighs risk	No cautionary statement	79464
116	Entacapone	Use if benefit outweighs risk	No cautionary statement	78665
116	Rasagiline mesilate	Use if benefit outweighs risk	No cautionary statement	49405
116	Zonisamide (indication: Parkinsonism)	Pregnancy-contraindicated	No cautionary statement	38508
116	Opicapone	Use if benefit outweighs risk	No cautionary statement	36958
116	Safinamide mesilate	Pregnancy-contraindicated	Mandatory contraceptive instruction	34866
116	Istradefylline	Pregnancy-contraindicated	No cautionary statement	28085
116	Levodopa	Use if benefit outweighs risk	No cautionary statement	19841
116	Pergolide mesilate	Use if benefit outweighs risk	No cautionary statement	1869
117	Etizolam	Use if benefit outweighs risk	No cautionary statement	89294363
117	Aripiprazole	Use if benefit outweighs risk	No cautionary statement	64906685
117	Sertraline hydrochloride	Use if benefit outweighs risk	No cautionary statement	63153298
117	Quetiapine fumarate	Use if benefit outweighs risk	No cautionary statement	50968022
117	Escitalopram oxalate	Use if benefit outweighs risk	No cautionary statement	49433991
117	Duloxetine hydrochloride	Use if benefit outweighs risk	No cautionary statement	44250447
117	Lithium carbonate	Pregnancy-contraindicated	No cautionary statement	43122063
117	Risperidone	Use if benefit outweighs risk	No cautionary statement	39023565
117	Trazodone hydrochloride	Use if benefit outweighs risk	No cautionary statement	30901467
117	Clotiazepam	Use if benefit outweighs risk	No cautionary statement	30508202
117	Paroxetine hydrochloride hydrate	Use if benefit outweighs risk	No cautionary statement	30392149
117	Fluvoxamine maleate	Use if benefit outweighs risk	No cautionary statement	25679407
117	Mirtazapine	Use if benefit outweighs risk	No cautionary statement	21925671
117	Olanzapine	Use if benefit outweighs risk	No cautionary statement	21783220
117	Atomoxetine hydrochloride	Use if benefit outweighs risk	No cautionary statement	21701412

117	Levomepromazine maleate	Use if benefit outweighs risk	No cautionary statement	21403547
117	Venlafaxine hydrochloride	Use if benefit outweighs risk	No cautionary statement	20223389
117	Amitriptyline hydrochloride	Use if benefit outweighs risk	No cautionary statement	15995520
117	Vortioxetine hydrobromide	Use if benefit outweighs risk	No cautionary statement	15581120
117	Lurasidone hydrochloride	Use if benefit outweighs risk	No cautionary statement	13767628
117	Chlorpromazine hydrochloride	Use if benefit outweighs risk	No cautionary statement	12240746
117	Brexpiprazole	Use if benefit outweighs risk	No cautionary statement	12000899
117	Methylphenidate hydrochloride	Use if benefit outweighs risk	No cautionary statement	11581317
117	Blonanserin	Use if benefit outweighs risk	No cautionary statement	11121643
117	Clomipramine hydrochloride	Use if benefit outweighs risk	No cautionary statement	8580453
117	Guanfacine hydrochloride	Pregnancy-contraindicated	No cautionary statement	7334690
117	Perospirone hydrochloride hydrate	Use if benefit outweighs risk	No cautionary statement	7236423
117	Haloperidol	Pregnancy-contraindicated	No cautionary statement	7043845
117	Amoxapine	Use if benefit outweighs risk	No cautionary statement	4434987
117	Hydroxyzine pamoate	Pregnancy-contraindicated	No cautionary statement	3845232
117	Paliperidone	Use if benefit outweighs risk	No cautionary statement	3669971
117	Zotepine	Use if benefit outweighs risk	No cautionary statement	3368128
117	Imipramine hydrochloride	Use if benefit outweighs risk	No cautionary statement	3331641
117	Milnacipran hydrochloride	Use if benefit outweighs risk	No cautionary statement	3258650
117	Nortriptyline hydrochloride	Use if benefit outweighs risk	No cautionary statement	2928839
117	Mianserin hydrochloride extract	Use if benefit outweighs risk	No cautionary statement	2814168
117	Asenapine maleate	Use if benefit outweighs risk	No cautionary statement	2580640
117	Pemoline	Use if benefit outweighs risk	No cautionary statement	2026053
117	Maprotiline hydrochloride	Use if benefit outweighs risk	No cautionary statement	2022348
117	Modafinil	Use if benefit outweighs risk	No cautionary statement	1964087
117	Hydroxyzine hydrochloride	Pregnancy-contraindicated	No cautionary statement	1731517

117	Propericiazine	Use if benefit outweighs risk	No cautionary statement	1520441
117	Perphenazine maleate	Use if benefit outweighs risk	No cautionary statement	1385059
117	Clozapine	Use if benefit outweighs risk	No cautionary statement	1122268
117	Prochlorperazine maleate	Use if benefit outweighs risk	No cautionary statement	1010554
117	Dosulepin hydrochloride	Use if benefit outweighs risk	No cautionary statement	956939
117	Sulpiride (200mg tablets)	Use if benefit outweighs risk	No cautionary statement	949528
117	Bromperidol	Pregnancy-contraindicated	No cautionary statement	918161
117	Setiptiline maleate	Use if benefit outweighs risk	No cautionary statement	601285
117	Fluphenazine maleate	Use if benefit outweighs risk	No cautionary statement	432132
117	Sultopride hydrochloride	Use if benefit outweighs risk	No cautionary statement	293401
117	Chlorpromazine phenolphthalinate	Use if benefit outweighs risk	No cautionary statement	254941
117	Lofepramine hydrochloride	Use if benefit outweighs risk	No cautionary statement	209167
117	Pipamperone hydrochloride	Use if benefit outweighs risk	No cautionary statement	200066
117	Lisdexamfetamine mesilate	Use if benefit outweighs risk	No cautionary statement	149856
117	Clocapramine hydrochloride hydrate	Use if benefit outweighs risk	No cautionary statement	64176
119	Lemborexant	Use if benefit outweighs risk	No cautionary statement	49635594
119	Pregabalin	Use if benefit outweighs risk	No cautionary statement	40870984
119	Mirogabalin besilate	Use if benefit outweighs risk	No cautionary statement	19396889
119	Suvorexant	Use if benefit outweighs risk	No cautionary statement	19146168
119	Ramelteon	Use if benefit outweighs risk	No cautionary statement	11325866
119	Acamprosate calcium	Use if benefit outweighs risk	No cautionary statement	1937926
119	Gabapentin enacarbil	Use if benefit outweighs risk	No cautionary statement	1379679
119	Dimethyl fumarate	Use if benefit outweighs risk	No cautionary statement	1244133
119	Piracetam	Use if benefit outweighs risk	No cautionary statement	1008336
119	Melatonin	Use if benefit outweighs risk	No cautionary statement	781000
119	Tiapride hydrochloride	Use if benefit outweighs risk	No cautionary statement	633430

119	Taltirelin hydrate	Use if benefit outweighs risk	No cautionary statement	583687
119	Lasmiditan succinate	Use if benefit outweighs risk	No cautionary statement	421005
119	Mazindol	Pregnancy-contraindicated	No cautionary statement	307357
119	Nalfurafine hydrochloride	Use if benefit outweighs risk	No cautionary statement	247263
119	Nalmefene hydrochloride hydrate	Use if benefit outweighs risk	No cautionary statement	119795
119	Riluzole	Pregnancy-contraindicated	No cautionary statement	87225
119	Tetrabenazine	Use if benefit outweighs risk	No cautionary statement	52842
119	Valbenazine tosilate	Use if benefit outweighs risk	No cautionary statement	44298
119	Memantine hydrochloride	Use if benefit outweighs risk	No cautionary statement	34248
119	Donepezil hydrochloride	Use if benefit outweighs risk	No cautionary statement	17684
119	Galantamine hydrobromide	Use if benefit outweighs risk	No cautionary statement	13845
122	Chlorphenesin carbamate	Use if benefit outweighs risk	No cautionary statement	4772969
122	Dantrolene sodium hydrate	Use if benefit outweighs risk	Contraindicated (Mandatory contraception)	1176724
122	Methocarbamol	Use if benefit outweighs risk	No cautionary statement	843119
123	Mepenzolate bromide	Use if benefit outweighs risk	No cautionary statement	5465516
123	Tiquizium bromide	Use if benefit outweighs risk	No cautionary statement	5350725
123	Propantheline bromide	Use if benefit outweighs risk	No cautionary statement	2459625
123	Pyridostigmine bromide	Use if benefit outweighs risk	No cautionary statement	1081095
123	Distigmine bromide	Use if benefit outweighs risk	No cautionary statement	592437
123	Mepenzolate bromide/Phenobarbital combination	Use if benefit outweighs risk	No cautionary statement	291408
123	Bethanechol chloride	Pregnancy-contraindicated	No cautionary statement	183968
123	Ambenonium chloride	Use if benefit outweighs risk	No cautionary statement	175616
123	Neostigmine bromide	Use if benefit outweighs risk	No cautionary statement	87664
129	Tafamidis meglumine	Use if benefit outweighs risk	Contraindicated (Mandatory contraception)	1284
212	Bisoprolol fumarate	Use if benefit outweighs risk	No cautionary statement	15907664

212	Propranolol hydrochloride (for Cardiovascular Diseases)	Use if benefit outweighs risk	No cautionary statement	11204749
212	Arotinolol hydrochloride	Pregnancy-contraindicated	No cautionary statement	2920862
212	Atenolol extract	Use if benefit outweighs risk	No cautionary statement	2808348
212	Mexiletine hydrochloride	Use if benefit outweighs risk	No cautionary statement	1663091
212	Pilsicainide hydrochloride hydrate	Use if benefit outweighs risk	No cautionary statement	1020719
212	Flecainide acetate	Pregnancy-contraindicated	No cautionary statement	577968
212	Cibenzoline succinate	Use if benefit outweighs risk	No cautionary statement	477148
212	Carteolol hydrochloride (rapid-release preparation)	Pregnancy-contraindicated	No cautionary statement	416060
212	Bepridil hydrochloride hydrate	Pregnancy-contraindicated	No cautionary statement	274704
212	Amiodarone hydrochloride	Use if benefit outweighs risk	No cautionary statement	272129
212	Aprindine hydrochloride	Pregnancy-contraindicated	No cautionary statement	195257
212	Disopyramide phosphate	Use if benefit outweighs risk	No cautionary statement	128956
212	Propafenone hydrochloride	Use if benefit outweighs risk	No cautionary statement	107382
212	Sotalol hydrochloride	Use if benefit outweighs risk	No cautionary statement	97844
212	Disopyramide	Use if benefit outweighs risk	No cautionary statement	81602
213	Isosorbide	Use if benefit outweighs risk	No cautionary statement	30243075
213	Spironolactone	Use if benefit outweighs risk	No cautionary statement	6625537
213	Furosemide	Use if benefit outweighs risk	No cautionary statement	6313467
213	Trichlormethiazide	Use if benefit outweighs risk	No cautionary statement	2940605
213	Azosemide	Use if benefit outweighs risk	No cautionary statement	1435500
213	Tolvaptan (other than 30mg tablets)	Pregnancy-contraindicated	Contraindicated (Mandatory contraception)	1226600
213	Acetazolamide (tablets)	Use if benefit outweighs risk	No cautionary statement	975673
213	Torasemide	Use if benefit outweighs risk	No cautionary statement	553103
213	Hydrochlorothiazide	Use if benefit outweighs risk	No cautionary statement	331054
213	Mefruside	Use if benefit outweighs risk	No cautionary statement	38200
213	Acetazolamide (powder)	Use if benefit outweighs risk	No cautionary statement	37261

213	Benzylhydrochlorothiazide	Use if benefit outweighs risk	No cautionary statement	21086
213	Triamterene	Use if benefit outweighs risk	No cautionary statement	9157
214	Olmesartan medoxomil	Pregnancy-contraindicated	Use if benefit outweighs risk (Contraceptive recommendation)	12860660
214	Telmisartan	Pregnancy-contraindicated	Use if benefit outweighs risk (Contraceptive recommendation)	10932196
214	Azilsartan	Pregnancy-contraindicated	Use if benefit outweighs risk (Contraceptive recommendation)	9762405
214	Candesartan cilexetil	Pregnancy-contraindicated	Use if benefit outweighs risk (Contraceptive recommendation)	8916317
214	Carvedilol	Use if benefit outweighs risk	No cautionary statement	7377994
214	Methyldopa hydrate	Use if benefit outweighs risk	No cautionary statement	5994944
214	Telmisartan/Amlodipine besilate combination	Pregnancy-contraindicated	Use if benefit outweighs risk (Contraceptive recommendation)	4903671
214	Irbesartan/Amlodipine besilate combination	Pregnancy-contraindicated	Use if benefit outweighs risk (Contraceptive recommendation)	4860598
214	Losartan potassium	Pregnancy-contraindicated	Use if benefit outweighs risk (Contraceptive recommendation)	4708192
214	Azilsartan/Amlodipine besilate combination	Pregnancy-contraindicated	Use if benefit outweighs risk (Contraceptive recommendation)	3955865
214	Cilnidipine	Pregnancy-contraindicated	No cautionary statement	3848388
214	Valsartan	Pregnancy-contraindicated	Use if benefit outweighs risk (Contraceptive	3702607

			recommendation)	
214	Enalapril maleate	Pregnancy-contraindicated	Use if benefit outweighs risk (Contraceptive recommendation)	3313587
214	Candesartan cilexetil/Amlodipine besilate combination	Pregnancy-contraindicated	Use if benefit outweighs risk (Contraceptive recommendation)	3301125
214	Urapidil	Use if benefit outweighs risk	No cautionary statement	3036499
214	Irbesartan	Pregnancy-contraindicated	Use if benefit outweighs risk (Contraceptive recommendation)	2904536
214	Doxazosin mesilate	Use if benefit outweighs risk	No cautionary statement	2658291
214	Esaxerenone	Use if benefit outweighs risk	No cautionary statement	2424191
214	Eplerenone	Use if benefit outweighs risk	No cautionary statement	2119340
214	Azelnidipine	Pregnancy-contraindicated	Use if benefit outweighs risk (Contraceptive recommendation)	1685449
214	Valsartan/Amlodipine besilate combination	Pregnancy-contraindicated	Use if benefit outweighs risk (Contraceptive recommendation)	1657827
214	Olmesartan medoxomil/Azelnidipine combination	Pregnancy-contraindicated	Use if benefit outweighs risk (Contraceptive recommendation)	1582588
214	Indapamide	Use if benefit outweighs risk	No cautionary statement	1419537
214	Telmisartan/Hydrochlorothiazide combination	Pregnancy-contraindicated	Use if benefit outweighs risk (Contraceptive recommendation)	841674
214	Metoprolol tartrate	Pregnancy-contraindicated	No cautionary statement	807118
214	Imidapril hydrochloride	Pregnancy-contraindicated	Use if benefit outweighs risk (Contraceptive	665711

			recommendati on)	
214	Candesartan cilexetil/Hydrochlorothiazide combination	Pregnancy-contraindicated	Use if benefit outweighs risk (Contraceptive recommendati on)	449280
214	Losartan potassium/Hydrochlorothiazide combination	Pregnancy-contraindicated	Use if benefit outweighs risk (Contraceptive recommendati on)	407410
214	Clonidine hydrochloride	Use if benefit outweighs risk	No cautionary statement	281854
214	Valsartan/Cilnidipine combination	Pregnancy-contraindicated	Use if benefit outweighs risk (Contraceptive recommendati on)	279447
214	Perindopril erbumine	Pregnancy-contraindicated	Use if benefit outweighs risk (Contraceptive recommendati on)	253656
214	Valsartan/Hydrochlorothiazide combination	Pregnancy-contraindicated	Use if benefit outweighs risk (Contraceptive recommendati on)	238026
214	Lisinopril hydrate	Pregnancy-contraindicated	Use if benefit outweighs risk (Contraceptive recommendati on)	172418
214	Irbesartan/Trichlormethiazide combination	Pregnancy-contraindicated	Use if benefit outweighs risk (Contraceptive recommendati on)	164509
214	Celiprolol hydrochloride	Pregnancy-contraindicated	No cautionary statement	156418
214	Prazosin hydrochloride	Use if benefit outweighs risk	No cautionary statement	134059
214	Efonidipine hydrochloride ethanolate	Pregnancy-contraindicated	No cautionary statement	131136
214	Betaxolol hydrochloride	Pregnancy-contraindicated	No cautionary statement	117960
214	Aliskiren fumarate	Pregnancy-contraindicated	Use if benefit outweighs risk (Contraceptive recommendati on)	98903

214	Nicardipine hydrochloride	Pregnancy-contraindicated	No cautionary statement	98379
214	Guanabenz acetate	Use if benefit outweighs risk	No cautionary statement	89047
214	Telmisartan/Amlodipine besilate/Hydrochlorothiazide combination	Pregnancy-contraindicated	Use if benefit outweighs risk (Contraceptive recommendation)	67407
214	Nilvadipine	Pregnancy-contraindicated	No cautionary statement	56575
214	Temocapril hydrochloride	Pregnancy-contraindicated	Use if benefit outweighs risk (Contraceptive recommendation)	56231
214	Bunazosin hydrochloride	Use if benefit outweighs risk	No cautionary statement	45998
214	Manidipine hydrochloride	Pregnancy-contraindicated	No cautionary statement	41343
214	Carteolol hydrochloride (extended-release preparation)	Pregnancy-contraindicated	No cautionary statement	30621
214	Terazosin hydrochloride hydrate	Use if benefit outweighs risk	No cautionary statement	21783
214	Bevantolol hydrochloride	Pregnancy-contraindicated	No cautionary statement	18437
214	Trandolapril	Pregnancy-contraindicated	Use if benefit outweighs risk (Contraceptive recommendation)	16243
214	Tripamide	Use if benefit outweighs risk	No cautionary statement	15238
214	Alacepril	Pregnancy-contraindicated	Use if benefit outweighs risk (Contraceptive recommendation)	10932
217	Amlodipine besilate	Use if benefit outweighs risk	No cautionary statement	56908346
217	Nifedipine	Use if benefit outweighs risk	No cautionary statement	17327093
217	Benidipine hydrochloride	Pregnancy-contraindicated	No cautionary statement	3449439
217	Verapamil hydrochloride	Pregnancy-contraindicated	No cautionary statement	3338149
217	Dilazep hydrochloride hydrate	Use if benefit outweighs risk	No cautionary statement	2709449
217	Diltiazem hydrochloride	Pregnancy-contraindicated	No cautionary statement	2474587
217	Dipyridamole	Use if benefit outweighs risk	No cautionary statement	2381375

217	Nicorandil	Use if benefit outweighs risk	No cautionary statement	2119956
217	Isoxsuprine hydrochloride	Pregnancy-contraindicated	No cautionary statement	1304989
217	Isosorbide mononitrate	Use if benefit outweighs risk	No cautionary statement	673603
217	Nitroglycerin	Use if benefit outweighs risk	No cautionary statement	222589
217	Isosorbide dinitrate (extended-release preparation)	Use if benefit outweighs risk	No cautionary statement	193718
217	Isosorbide dinitrate (rapid-release preparation)	Use if benefit outweighs risk	No cautionary statement	40263
217	Trapidil	Use if benefit outweighs risk	No cautionary statement	12761
217	Nitrendipine	Pregnancy-contraindicated	No cautionary statement	6986
217	Trimetazidine hydrochloride	Use if benefit outweighs risk	No cautionary statement	1200
218	Rosuvastatin calcium	Pregnancy-contraindicated	No cautionary statement	37244366
218	Atorvastatin calcium hydrate	Pregnancy-contraindicated	No cautionary statement	17227943
218	Pitavastatin calcium hydrate	Pregnancy-contraindicated	No cautionary statement	15767007
218	Pemafibrate	Pregnancy-contraindicated	No cautionary statement	14336561
218	Ezetimibe	Use if benefit outweighs risk	No cautionary statement	6410517
218	Pravastatin sodium	Pregnancy-contraindicated	No cautionary statement	5837825
218	Bezafibrate	Pregnancy-contraindicated	No cautionary statement	5704794
218	Colestimide	No cautionary statement	No cautionary statement	5126692
218	Fenofibrate	Pregnancy-contraindicated	No cautionary statement	3823337
218	Omega-3-acid ethyl esters	Use if benefit outweighs risk	No cautionary statement	3171669
218	Polyenephosphatidylcholine	No cautionary statement	No cautionary statement	1925337
218	Ezetimibe/Rosuvastatin calcium combination	Pregnancy-contraindicated	No cautionary statement	1253468
218	Simvastatin	Pregnancy-contraindicated	No cautionary statement	820407
218	Fluvastatin sodium	Pregnancy-contraindicated	No cautionary statement	662102
218	Ezetimibe/Atorvastatin calcium hydrate combination	Pregnancy-contraindicated	No cautionary statement	658889
218	Probucol	Pregnancy-contraindicated	No cautionary statement	420211
218	Colestyramine	Use if benefit outweighs risk	No cautionary statement	129441

218	Niceritrol	Use if benefit outweighs risk	No cautionary statement	54802
218	Pitavastatin calcium hydrate/Ezetimibe combination	Pregnancy-contraindicated	No cautionary statement	37131
218	Elastase ES	No cautionary statement	No cautionary statement	35856
218	Dextran sulfate sodium sulfur 18	Use if benefit outweighs risk	No cautionary statement	32188
218	Nicomol	Use if benefit outweighs risk	No cautionary statement	18648
219	Lomerizine hydrochloride	Pregnancy-contraindicated	No cautionary statement	33273563
219	Tocopherol acetate	Use if benefit outweighs risk	No cautionary statement	29854376
219	Ferric citrate hydrate	Use if benefit outweighs risk	No cautionary statement	8184577
219	Precipitated calcium carbonate (indication: hyperphosphatemia)	No cautionary statement	No cautionary statement	3480103
219	Lanthanum carbonate hydrate	Use if benefit outweighs risk	No cautionary statement	3066755
219	Amezinium metilsulfate	Use if benefit outweighs risk	No cautionary statement	3047921
219	Sacubitril valsartan sodium hydrate	Pregnancy-contraindicated	Use if benefit outweighs risk (Contraceptive recommendation)	2130089
219	Sevelamer hydrochloride	Use if benefit outweighs risk	No cautionary statement	1456823
219	Bixalomer	Use if benefit outweighs risk	No cautionary statement	1266185
219	Sodium polystyrene sulfonate	Use if benefit outweighs risk	No cautionary statement	1087466
219	Sucroferric oxyhydroxide	Use if benefit outweighs risk	No cautionary statement	1026964
219	Selexipag	Use if benefit outweighs risk	No cautionary statement	914943
219	Calcium Polystyrene sulfonate	Use if benefit outweighs risk	No cautionary statement	812926
219	Ifenprodil tartrate	Use if benefit outweighs risk	No cautionary statement	786261
219	Nicergoline	Use if benefit outweighs risk	No cautionary statement	466041
219	Bosentan hydrate	Pregnancy-contraindicated	Contraindicated (Mandatory contraception)	464132
219	Beraprost (indication: pulmonary arterial pulmonary hypertension)	Pregnancy-contraindicated	No cautionary statement	438310
219	Tadalafil (indication: Arterial Pulmonary Hypertension)	Use if benefit outweighs risk	No cautionary statement	428534
219	Amlodipine besilate/Atorvastatin calcium hydrate combination No.3	Pregnancy-contraindicated	No cautionary statement	405538

219	Amlodipine besilate/Atorvastatin calcium hydrate combination No.4	Pregnancy-contraindicated	No cautionary statement	366141
219	Sildenafil citrate	Use if benefit outweighs risk	No cautionary statement	324508
219	Riociguat	Pregnancy-contraindicated	Contraindicated (Mandatory contraception)	292360
219	Ivabradine hydrochloride	Pregnancy-contraindicated	Contraindicated (Mandatory contraception)	265434
219	Amlodipine besilate/Atorvastatin calcium hydrate combination No.1	Pregnancy-contraindicated	No cautionary statement	112833
219	Sodium zirconium cyclosilicate hydrate	Use if benefit outweighs risk	No cautionary statement	67042
219	Amlodipine besilate/Atorvastatin calcium hydrate combination No.2	Pregnancy-contraindicated	No cautionary statement	22150
232	Rebamipide	Use if benefit outweighs risk	No cautionary statement	202756647
232	Famotidine	Use if benefit outweighs risk	No cautionary statement	56950092
232	Vonoprazan fumarate	Use if benefit outweighs risk	No cautionary statement	31844116
232	Sodium alginate	No cautionary statement	No cautionary statement	31490039
232	Teprenone	Use if benefit outweighs risk	No cautionary statement	30272993
232	Esomeprazole magnesium hydrate	Use if benefit outweighs risk	No cautionary statement	28982812
232	Sulpiride (50mg tablets)	Use if benefit outweighs risk	No cautionary statement	23327548
232	Lansoprazole	Use if benefit outweighs risk	No cautionary statement	20262796
232	Rabeprazole sodium	Use if benefit outweighs risk	No cautionary statement	13745891
232	Polaprezinc	Use if benefit outweighs risk	No cautionary statement	10213472
232	Lafutidine	Use if benefit outweighs risk	No cautionary statement	8903290
232	Sucralfate hydrate	Use if benefit outweighs risk	No cautionary statement	7272056
232	L-glutamine (tablets)	Use if benefit outweighs risk	No cautionary statement	6876475
232	Irsogladine maleate	Use if benefit outweighs risk	No cautionary statement	5929853
232	Nizatidine	Use if benefit outweighs risk	No cautionary statement	3637654
232	Aldioxa	Use if benefit outweighs risk	No cautionary statement	3123809
232	Troxipide	Use if benefit outweighs risk	No cautionary statement	2687914
232	Cimetidine	Use if benefit outweighs risk	No cautionary statement	2536035

232	Omeprazole	Use if benefit outweighs risk	No cautionary statement	2518456
232	Ecabet sodium hydrate	Use if benefit outweighs risk	No cautionary statement	2061316
232	Sofalcone	Use if benefit outweighs risk	No cautionary statement	925053
232	Roxatidine acetate hydrochloride	Use if benefit outweighs risk	No cautionary statement	692733
232	Dicyclomine hydrochloride/Dried aluminum Hydroxide Gel/Magnesium oxide combination	Pregnancy-contraindicated	No cautionary statement	632081
232	Methylmethionine sulfonium chloride (tablets)	Use if benefit outweighs risk	No cautionary statement	416720
232	Cetraxate hydrochloride	Use if benefit outweighs risk	No cautionary statement	384805
232	Misoprostol	Use if benefit outweighs risk	Contraindicated (Mandatory contraception)	365252
232	Sodium gualenate hydrate	No cautionary statement	No cautionary statement	252035
232	Benexate hydrochloride betadex	Pregnancy-contraindicated	No cautionary statement	153521
232	Pirenzepine hydrochloride hydrate	Use if benefit outweighs risk	No cautionary statement	141563
232	Methylmethionine sulfonium chloride (powder)	Use if benefit outweighs risk	No cautionary statement	82853
232	L-glutamine (granules)	Use if benefit outweighs risk	No cautionary statement	74622
232	Egualen sodium hydrate	Use if benefit outweighs risk	No cautionary statement	30751
235	Sennoside A/B (tablets)	Use if benefit outweighs risk	No cautionary statement	24550021
235	Sodium picosulfate hydrate	Use if benefit outweighs risk	No cautionary statement	12939760
235	Macrogol 4000	Use if benefit outweighs risk	No cautionary statement	9015321
235	Elobixibat hydrate	Use if benefit outweighs risk	No cautionary statement	7850588
235	Lubiprostone	Pregnancy-contraindicated	No cautionary statement	6974951
235	Sennoside A/B (granules)	Use if benefit outweighs risk	No cautionary statement	3439904
235	Powdered coptis rhizome/Powdered senna leaf/Powdered rhubarb/Magnesium sulfate hydrate/Magnesium oxide combination	Use if benefit outweighs risk	No cautionary statement	2729969
235	Senna extract	Use if benefit outweighs risk	No cautionary statement	2147862
235	Casanthranol/Dioctyl sodium sulfosuccinate combination	Use if benefit outweighs risk	No cautionary statement	1423094
235	Naldemedine tosilate	Use if benefit outweighs risk	No cautionary statement	537341
235	Magnesium sulfate hydrate	Use if benefit outweighs risk	No cautionary statement	142440

235	Carmellose	Use if benefit outweighs risk	No cautionary statement	86215
235	Castor oil	Use if benefit outweighs risk	No cautionary statement	38868
235	Castor oil (improved flavor)	Use if benefit outweighs risk	No cautionary statement	6500
235	Compound glycyrrhiza	Use if benefit outweighs risk	No cautionary statement	3426
236	Ursodeoxycholic acid	Use if benefit outweighs risk	No cautionary statement	46414614
236	Trepibutone	Use if benefit outweighs risk	No cautionary statement	135303
236	Chenodeoxycholic acid	Pregnancy-contraindicated	No cautionary statement	20302
239	Mesalazine	Use if benefit outweighs risk	No cautionary statement	76612059
239	Mosapride citrate hydrate	Use if benefit outweighs risk	No cautionary statement	58408500
239	Domperidone	Pregnancy-contraindicated	No cautionary statement	37156699
239	Polycarbophil calcium	Use if benefit outweighs risk	No cautionary statement	26634447
239	Metoclopramide	Use if benefit outweighs risk	No cautionary statement	25246732
239	Trimebutine maleate	No cautionary statement	No cautionary statement	19089570
239	Acotiamide hydrochloride hydrate	Use if benefit outweighs risk	No cautionary statement	12485043
239	Ramosetron hydrochloride	Use if benefit outweighs risk	No cautionary statement	6211871
239	Linaclotide	Use if benefit outweighs risk	No cautionary statement	6192607
239	Itopride hydrochloride	Use if benefit outweighs risk	No cautionary statement	5458663
239	Cevimeline hydrochloride hydrate	Use if benefit outweighs risk	No cautionary statement	1437965
239	Pilocarpine hydrochloride	Use if benefit outweighs risk	No cautionary statement	1174851
239	Carotegrast methyl	Pregnancy-contraindicated	Contraindicated (Mandatory contraception)	639420
239	Budesonide	Use if benefit outweighs risk	No cautionary statement	282794
244	Metenolone acetate	Pregnancy-contraindicated	No cautionary statement	56005
247	Dydrogesterone	No cautionary statement	No cautionary statement	15731963
247	Conjugated estrogens	Pregnancy-contraindicated	No cautionary statement	12232477
247	Medroxyprogesterone acetate	Pregnancy-contraindicated	No cautionary statement	7237990
247	Chlormadinone acetate	No cautionary statement	No cautionary statement	5324040

247	Estradiol	Pregnancy-contraindicated	No cautionary statement	5038172
247	Norethisterone	Pregnancy-contraindicated	No cautionary statement	1711273
247	Progesterone	No cautionary statement	No cautionary statement	1418636
247	Estriol	Pregnancy-contraindicated	No cautionary statement	291618
247	Ethinylestradiol	No cautionary statement	No cautionary statement	1385
248	Drospirenone/Ethinylestradiol betadex combination	Pregnancy-contraindicated	Contraindicated (Mandatory contraception)	62798390
248	Norethisterone/Ethinylestradiol combination	Pregnancy-contraindicated	Contraindicated (Mandatory contraception)	52960625
248	Levonorgestrel/Ethinylestradiol combination	Pregnancy-contraindicated	Contraindicated (Mandatory contraception)	7266171
248	Norgestrel/Ethinylestradiol combination	Pregnancy-contraindicated	No cautionary statement	3382445
248	Estradiol/Levonorgestrel combination	Pregnancy-contraindicated	No cautionary statement	278811
249	Dienogest	Pregnancy-contraindicated	No cautionary statement	120798129
249	Kallidinogenase	Use if benefit outweighs risk	No cautionary statement	9618959
249	Relugolix	Pregnancy-contraindicated	No cautionary statement	6217903
249	Semaglutide	Pregnancy-contraindicated	Contraindicated (Mandatory contraception)	5182797
249	Clomifene citrate	Pregnancy-contraindicated	No cautionary statement	1334133
249	Tolvaptan (30mg tablets)	Pregnancy-contraindicated	No cautionary statement	775942
249	Cyclofenil	Pregnancy-contraindicated	No cautionary statement	748260
249	Danazol	Pregnancy-contraindicated	No cautionary statement	130469
249	Mitotane	Use if benefit outweighs risk	Contraindicated (Mandatory contraception)	37723
249	Osilodrostat phosphate	Pregnancy-contraindicated	Contraindicated (Mandatory contraception)	13899
249	Mepitiostane	Pregnancy-contraindicated	No cautionary statement	2589
249	Dinoprostone	Use if benefit outweighs risk	No cautionary statement	<1000
249	Dutasteride	Pregnancy-contraindicated	No cautionary statement	<1000

249	Trilostane	Pregnancy-contraindicated	Contraindicated (Mandatory contraception)	<1000
253	Methylergometrine maleate	Pregnancy-contraindicated	No cautionary statement	1226770
259	Ritodrine hydrochloride	Pregnancy-contraindicated	No cautionary statement	8461537
259	Vibegron	Use if benefit outweighs risk	No cautionary statement	2897669
259	Quercus salicina extract	No cautionary statement	No cautionary statement	2095954
259	Solifenacin succinate	Use if benefit outweighs risk	No cautionary statement	2084509
259	Mirabegron	Pregnancy-contraindicated	Use if benefit outweighs risk (Contraceptive recommendation)	1618575
259	Imidafenacin	Use if benefit outweighs risk	No cautionary statement	1153737
259	Fesoterodine fumarate	Use if benefit outweighs risk	No cautionary statement	981996
259	Propiverine hydrochloride	Use if benefit outweighs risk	No cautionary statement	873574
259	Flavoxate hydrochloride	Use if benefit outweighs risk	No cautionary statement	774006
259	Oxybutynin hydrochloride	Use if benefit outweighs risk	No cautionary statement	473957
259	Tolterodine tartrate	Use if benefit outweighs risk	No cautionary statement	38778
259	Cernitin pollen extract	No cautionary statement	No cautionary statement	2606
259	Elsamet S combination (for enlarged prostate)	No cautionary statement	No cautionary statement	<1000
259	Naftopidil	No cautionary statement	No cautionary statement	<1000
259	Silodosin	No cautionary statement	No cautionary statement	<1000
259	Tadalafil	No cautionary statement	No cautionary statement	<1000
259	Tamsulosin hydrochloride	No cautionary statement	No cautionary statement	<1000
311	Alfacalcidol	Use if benefit outweighs risk	No cautionary statement	14182968
311	Eldecalcitol	Pregnancy-contraindicated	Contraindicated (Mandatory contraception)	8062335
311	Vitamin A oil	Pregnancy-contraindicated	No cautionary statement	2036711
311	Calcitriol	Use if benefit outweighs risk	No cautionary statement	1063989
311	Etretinate	Pregnancy-contraindicated	Use if benefit outweighs risk	140776

			(Contraceptive recommendation)	
311	Falecalcitriol	No cautionary statement	No cautionary statement	31747
311	Retinol palmitate	Pregnancy-contraindicated	No cautionary statement	<1000
317	Ascorbic acid/Calcium pantothenate combination	No cautionary statement	No cautionary statement	262523410
317	Riboflavin/Pyridoxine hydrochloride combination	Use if benefit outweighs risk	No cautionary statement	46474850
317	Benfotiamine/Pyridoxine hydrochloride (25mg)/Cyanocobalamin combination	No cautionary statement	No cautionary statement	8394876
317	Octotiamine/Riboflavin/Pyridoxine hydrochloride/Cyanocobalamin combination	No cautionary statement	No cautionary statement	7687664
317	Fursultiamine hydrochloride/Pyridoxal phosphate hydrate/Hydroxocobalamin acetate combination	No cautionary statement	No cautionary statement	4629536
317	Benfotiamine/Pyridoxine hydrochloride (50mg)/Cyanocobalamin combination	No cautionary statement	No cautionary statement	1670590
317	Panvitan (multivitamin)	Pregnancy-contraindicated	No cautionary statement	781519
317	Thiamine disulfide/Pyridoxine hydrochloride/Cyanocobalamin combination	No cautionary statement	No cautionary statement	135932
317	Calcium pantothenate/Riboflavin/Pyridoxine hydrochloride combination	No cautionary statement	No cautionary statement	105943
317	Thiamine disulfide/Pyridoxine hydrochloride/Cyanocobalamin combination	No cautionary statement	No cautionary statement	69894
317	Benfotiamine/Pyridoxine hydrochloride/Cyanocobalamin combination (Powder)	No cautionary statement	No cautionary statement	33385
325	ES-polytamin combination (essential amino acid preparation)	Use if benefit outweighs risk	No cautionary statement	3951640
325	Racol-NF semisolid for enteral Use	Use if benefit outweighs risk	No cautionary statement	2807069
325	Elental combination (enteral formula)	Pregnancy-contraindicated	No cautionary statement	1503236
325	Ensure liquid (enteral formula)	Pregnancy-contraindicated	No cautionary statement	886004
325	Enevo combination (enteral formula)	Use if benefit outweighs risk	No cautionary statement	792885
325	Livact combination (branched-chain amino acid preparation)	Use if benefit outweighs risk	No cautionary statement	755774
325	Ensure H (enteral formula)	Pregnancy-contraindicated	No cautionary statement	689153
325	Enoras liquid (enteral formula)	Use if benefit outweighs risk	No cautionary statement	395828
325	Twinline-NF liquid (enteral formula)	Use if benefit outweighs risk	No cautionary statement	122114

325	Aminoleban EN (oral nutrition for liver failure)	Use if benefit outweighs risk	No cautionary statement	113857
325	Elental P combination (enteral formula)	Pregnancy-contraindicated	No cautionary statement	64242
325	Amiyu combination (essential amino acids for renal failure)	Use if benefit outweighs risk	No cautionary statement	19269
325	Hepan ED combination (nutrients for hepatic insufficiency)	Use if benefit outweighs risk	No cautionary statement	6057
333	Warfarin potassium	Pregnancy-contraindicated	No cautionary statement	8536447
333	Apixaban	Use if benefit outweighs risk	No cautionary statement	1291686
333	Edoxaban tosilate hydrate	Use if benefit outweighs risk	No cautionary statement	1189796
333	Rivaroxaban	Pregnancy-contraindicated	No cautionary statement	643197
333	Dabigatran etexilate methanesulfonate	Use if benefit outweighs risk	No cautionary statement	227777
339	Ethyl icosapentate	Use if benefit outweighs risk	No cautionary statement	8451627
339	Limaprost alfadex	Pregnancy-contraindicated	No cautionary statement	7792499
339	Aspirin	Pregnancy-contraindicated	No cautionary statement	7044988
339	Beraprost	Pregnancy-contraindicated	No cautionary statement	2903566
339	Clopidogrel sulfate	Use if benefit outweighs risk	No cautionary statement	2305442
339	Cilostazol	Pregnancy-contraindicated	No cautionary statement	2099325
339	Sarpogrelate hydrochloride	Pregnancy-contraindicated	No cautionary statement	1214408
339	Aspirin/aluminum glycinate/Magnesium carbonate	Pregnancy-contraindicated	No cautionary statement	876708
339	Aspirin/Lansoprazole	Pregnancy-contraindicated	No cautionary statement	504166
339	Aspirin/Vonoprazan fumarate combination	Pregnancy-contraindicated	No cautionary statement	302617
339	Prasugrel hydrochloride	Use if benefit outweighs risk	No cautionary statement	297171
339	Ticlopidine hydrochloride	Use if benefit outweighs risk	No cautionary statement	100873
339	Clopidogrel sulfate/Aspirin combination	Pregnancy-contraindicated	No cautionary statement	3465
392	Glutathione	No cautionary statement	No cautionary statement	4664201
392	Zinc acetate hydrate	Use if benefit outweighs risk	No cautionary statement	3183105
392	Spherical adsorption charcoal	Use if benefit outweighs risk	No cautionary statement	1551029
392	Penicillamine	Pregnancy-contraindicated	No cautionary statement	251852

392	Trientine hydrochloride	Use if benefit outweighs risk	No cautionary statement	210064
392	Deferasirox	Use if benefit outweighs risk	No cautionary statement	58756
392	Calcium folinate hydrate	Pregnancy-contraindicated	No cautionary statement	34516
392	Cysteamine bitartrate	Use if benefit outweighs risk	Use if benefit outweighs risk (Contraceptive recommendation)	13317
392	Acetylcysteine	Use if benefit outweighs risk	No cautionary statement	<1000
392	Calcium sodium edetate hydrate	No cautionary statement	No cautionary statement	<1000
393	Cyanamide	Pregnancy-contraindicated	No cautionary statement	697924
393	Disulfiram	Pregnancy-contraindicated	No cautionary statement	31060
394	Febuxostat	Use if benefit outweighs risk	No cautionary statement	6604852
394	Colchicine	Pregnancy-contraindicated	No cautionary statement	3230798
394	Potassium citrate/Sodium citrate hydrate combination	No cautionary statement	No cautionary statement	2715261
394	Allopurinol	Use if benefit outweighs risk	No cautionary statement	1662901
394	Topiroxostat	Use if benefit outweighs risk	No cautionary statement	797789
394	Dotinurad	Use if benefit outweighs risk	No cautionary statement	411389
394	Benzbromarone	Pregnancy-contraindicated	No cautionary statement	307572
394	Probenecid	Use if benefit outweighs risk	No cautionary statement	52400
396	Metformin hydrochloride	Pregnancy-contraindicated	No cautionary statement	74859586
396	Dapagliflozin propylene glycolate hydrate	Pregnancy-contraindicated	No cautionary statement	7505221
396	Vildagliptin/Metformin hydrochloride combination	Pregnancy-contraindicated	No cautionary statement	6852092
396	Empagliflozin	Pregnancy-contraindicated	No cautionary statement	6202787
396	Voglibose	Use if benefit outweighs risk	No cautionary statement	6173053
396	Sitagliptin phosphate hydrate	Use if benefit outweighs risk	No cautionary statement	6126607
396	Glimepiride	Pregnancy-contraindicated	No cautionary statement	4955282
396	Miglitol	Pregnancy-contraindicated	No cautionary statement	4189909
396	Ipragliflozin L-proline	Pregnancy-contraindicated	No cautionary statement	4128367

396	Repaglinide	Pregnancy-contraindicated	No cautionary statement	3035911
396	Canagliflozin hydrate	Pregnancy-contraindicated	No cautionary statement	2730372
396	Luseogliflozin hydrate	Pregnancy-contraindicated	No cautionary statement	2598036
396	Tofogliflozin hydrate	Pregnancy-contraindicated	No cautionary statement	2344129
396	Vildagliptin	Use if benefit outweighs risk	No cautionary statement	2191557
396	Pioglitazone hydrochloride	Pregnancy-contraindicated	No cautionary statement	2168042
396	Linagliptin	Use if benefit outweighs risk	No cautionary statement	2104513
396	Teneligliptin hydrobromide hydrate	Use if benefit outweighs risk	No cautionary statement	1985455
396	Imeglimin hydrochloride	Pregnancy-contraindicated	No cautionary statement	1943118
396	Mitiglinide calcium hydrate	Pregnancy-contraindicated	No cautionary statement	1607201
396	Empagliflozin/Linagliptin combination	Pregnancy-contraindicated	No cautionary statement	1590513
396	Teneligliptin hydrobromide hydrate/Canagliflozin hydrate combination	Pregnancy-contraindicated	No cautionary statement	1427177
396	Mitiglinide calcium hydrate/Voglibose combination	Pregnancy-contraindicated	No cautionary statement	1398988
396	Sitagliptin phosphate hydrate/Ipragliflozin L-proline combination	Pregnancy-contraindicated	No cautionary statement	1263429
396	Anagliptin/Metformin hydrochloride combination	Pregnancy-contraindicated	No cautionary statement	1234945
396	Alogliptin benzoate/Metformin hydrochloride combination	Pregnancy-contraindicated	No cautionary statement	866588
396	Gliclazide	Pregnancy-contraindicated	No cautionary statement	848905
396	Alogliptin benzoate	Use if benefit outweighs risk	No cautionary statement	798294
396	Anagliptin	Use if benefit outweighs risk	No cautionary statement	696192
396	Saxagliptin hydrate	Use if benefit outweighs risk	No cautionary statement	672038
396	Acarbose	Pregnancy-contraindicated	No cautionary statement	600155
396	Glibenclamide	Pregnancy-contraindicated	No cautionary statement	226825
396	Nateglinide	Pregnancy-contraindicated	No cautionary statement	220569
396	Alogliptin benzoate/Pioglitazone hydrochloride combination	Pregnancy-contraindicated	No cautionary statement	161810
396	Pioglitazone hydrochloride/Metformin hydrochloride combination	Pregnancy-contraindicated	No cautionary statement	133409
396	Buformin hydrochloride	Pregnancy-contraindicated	No cautionary statement	52512

396	Pioglitazone hydrochloride/Glimepiride combination	Pregnancy-contraindicated	No cautionary statement	27562
396	Omarigliptin	Use if benefit outweighs risk	No cautionary statement	26813
396	Trelagliptin succinate	Use if benefit outweighs risk	No cautionary statement	14906
399	Adenosine triphosphate disodium hydrate	Use if benefit outweighs risk	No cautionary statement	50913541
399	L-cysteine	No cautionary statement	No cautionary statement	46596404
399	Tacrolimus hydrate	Use if benefit outweighs risk	No cautionary statement	20709517
399	Mycophenolate mofetil	Pregnancy-contraindicated	Contraindicated (Mandatory contraception)	16630384
399	Methotrexate (indication: rheumatism)	Pregnancy-contraindicated	Contraindicated (Mandatory contraception)	9273876
399	Ciclosporin	Use if benefit outweighs risk	No cautionary statement	9091671
399	Lactulose	Use if benefit outweighs risk	No cautionary statement	8683561
399	Hydroxychloroquine sulfate	Use if benefit outweighs risk	Use if benefit outweighs risk (Contraceptive recommendation)	7118749
399	Levocarnitine (liquid)	Use if benefit outweighs risk	No cautionary statement	6137396
399	Iguratimod	Pregnancy-contraindicated	No cautionary statement	5827885
399	Azathioprine	Use if benefit outweighs risk	Use if benefit outweighs risk (Contraceptive recommendation)	4966942
399	Camostat mesilate	Use if benefit outweighs risk	No cautionary statement	3609786
399	Mizoribine	Pregnancy-contraindicated	No cautionary statement	2355649
399	Apremilast	Pregnancy-contraindicated	Contraindicated (Mandatory contraception)	1096640
399	Everolimus (indication: rejection after organ transplantation)	Pregnancy-contraindicated	No cautionary statement	1076330
399	L-arginine hydrochloride/L-arginine combination	Use if benefit outweighs risk	No cautionary statement	997253
399	Alendronate sodium hydrate	Use if benefit outweighs risk	Use if benefit outweighs risk (Contraceptive recommendation)	827607

399	Tofacitinib citrate	Pregnancy-contraindicated	Contraindicated (Mandatory contraception)	760174
399	Eltrombopag olamine	Use if benefit outweighs risk	Contraindicated (Mandatory contraception)	698151
399	Baricitinib	Pregnancy-contraindicated	Contraindicated (Mandatory contraception)	693976
399	Evocalcet	Pregnancy-contraindicated	No cautionary statement	683582
399	Bazedoxifene acetate	Pregnancy-contraindicated	No cautionary statement	599220
399	Upadacitinib hydrate	Pregnancy-contraindicated	Contraindicated (Mandatory contraception)	557057
399	Epalrestat	Use if benefit outweighs risk	No cautionary statement	458995
399	Raloxifene hydrochloride	Pregnancy-contraindicated	No cautionary statement	423349
399	Daprodustat	Use if benefit outweighs risk	No cautionary statement	294409
399	Fingolimod hydrochloride	Pregnancy-contraindicated	Contraindicated (Mandatory contraception)	257937
399	Lactitol hydrate	Use if benefit outweighs risk	No cautionary statement	254073
399	Filgotinib maleate	Pregnancy-contraindicated	Contraindicated (Mandatory contraception)	225233
399	Sodium risedronate hydrate	Pregnancy-contraindicated	Use if benefit outweighs risk (Contraceptive recommendation)	204302
399	Sodium phenylbutyrate	Use if benefit outweighs risk	No cautionary statement	196686
399	Nintedanib ethanesulfonate	Pregnancy-contraindicated	Contraindicated (Mandatory contraception)	186759
399	Levocarnitine (tablets)	Use if benefit outweighs risk	No cautionary statement	179057
399	Leflunomide	Pregnancy-contraindicated	Contraindicated (Mandatory contraception)	167487
399	Siponimod fumaric acid	Pregnancy-contraindicated	Contraindicated (Mandatory contraception)	96996
399	Peficitinib hydrobromide	Pregnancy-contraindicated	Contraindicated (Mandatory contraception)	66223
399	Cinacalcet hydrochloride	Use if benefit outweighs risk	No cautionary statement	65230

399	Sapropterin hydrochloride	Use if benefit outweighs risk	No cautionary statement	64692
399	Diazoxide	Use if benefit outweighs risk	No cautionary statement	51388
399	Minodronic acid hydrate	Pregnancy-contraindicated	No cautionary statement	48728
399	Inosine pranobex	Use if benefit outweighs risk	No cautionary statement	48301
399	Roxadustat	Pregnancy-contraindicated	Contraindicated (Mandatory contraception)	44496
399	Vadadustat	Use if benefit outweighs risk	No cautionary statement	39659
399	Betaine	Use if benefit outweighs risk	No cautionary statement	33697
399	Pirfenidone	Use if benefit outweighs risk	No cautionary statement	26974
399	Avacopan	Use if benefit outweighs risk	No cautionary statement	25958
399	Molidustat sodium	Pregnancy-contraindicated	Contraindicated (Mandatory contraception)	13823
399	Anamorelin hydrochloride	Use if benefit outweighs risk	No cautionary statement	12766
399	Enarodustat	Use if benefit outweighs risk	Contraindicated (Mandatory contraception)	11799
399	Carglumic acid	Use if benefit outweighs risk	No cautionary statement	9890
399	Ibandronate sodium hydrate	Pregnancy-contraindicated	No cautionary statement	5696
399	Ipriflavone	Use if benefit outweighs risk	No cautionary statement	2386
399	Etidronate disodium	Pregnancy-contraindicated	No cautionary statement	1608
421	Temozolomide	Pregnancy-contraindicated	Contraindicated (Mandatory contraception)	63647
421	Cyclophosphamide hydrate	Use if benefit outweighs risk	Contraindicated (Mandatory contraception)	46204
421	Busulfan	Use if benefit outweighs risk	No cautionary statement	<1000
421	Estramustine phosphate sodium hydrate	No cautionary statement	No cautionary statement	<1000
421	Melphalan	Use if benefit outweighs risk	Contraindicated (Mandatory contraception)	<1000
422	Capecitabine	Pregnancy-contraindicated	Contraindicated (Mandatory contraception)	1845567
422	Tegafur/Gimeracil/Oteracil potassium combination	Pregnancy-contraindicated	No cautionary statement	884873

422	Tegafur/Uracil combination	Pregnancy-contraindicated	No cautionary statement	239649
422	Hydroxycarbamide	Pregnancy-contraindicated	Contraindicated (Mandatory contraception)	161818
422	Mercaptopurine hydrate	Use if benefit outweighs risk	No cautionary statement	106533
422	Methotrexate	Use if benefit outweighs risk	No cautionary statement	88972
422	Doxifluridine	Use if benefit outweighs risk	No cautionary statement	23209
422	Cytarabine ocfosfate hydrate	Use if benefit outweighs risk	No cautionary statement	<1000
422	Fludarabine phosphate	Pregnancy-contraindicated	Contraindicated (Mandatory contraception)	<1000
424	Etoposide	Pregnancy-contraindicated	Contraindicated (Mandatory contraception)	3259
429	Tamoxifen citrate	Pregnancy-contraindicated	Contraindicated (Mandatory contraception)	20221476
429	Letrozole	Pregnancy-contraindicated	No cautionary statement	1276746
429	Abemaciclib	Use if benefit outweighs risk	No cautionary statement	1134098
429	Anastrozole	Pregnancy-contraindicated	No cautionary statement	883730
429	Olaparib	Use if benefit outweighs risk	Contraindicated (Mandatory contraception)	819786
429	Palbociclib	Pregnancy-contraindicated	Contraindicated (Mandatory contraception)	389560
429	Toremifene citrate	Pregnancy-contraindicated	No cautionary statement	363360
429	Alectinib hydrochloride	Pregnancy-contraindicated	Contraindicated (Mandatory contraception)	339970
429	Dasatinib hydrate	Pregnancy-contraindicated	Contraindicated (Mandatory contraception)	331974
429	Anagrelide hydrochloride hydrate	Use if benefit outweighs risk	No cautionary statement	329961
429	Nilotinib hydrochloride hydrate	Pregnancy-contraindicated	Contraindicated (Mandatory contraception)	302369
429	Imatinib mesilate	Pregnancy-contraindicated	Contraindicated (Mandatory contraception)	281455
429	Exemestane	Pregnancy-contraindicated	No cautionary statement	247088

429	Everolimus	Pregnancy-contraindicated	Contraindicated (Mandatory contraception)	144075
429	Ruxolitinib phosphate	Pregnancy-contraindicated	Contraindicated (Mandatory contraception)	96968
429	Osimertinib mesilate	Pregnancy-contraindicated	Contraindicated (Mandatory contraception)	87972
429	Bosutinib hydrate	Pregnancy-contraindicated	Contraindicated (Mandatory contraception)	86457
429	Pazopanib hydrochloride	Pregnancy-contraindicated	Contraindicated (Mandatory contraception)	77184
429	Niraparib tosilate hydrate	Use if benefit outweighs risk	Contraindicated (Mandatory contraception)	69941
429	Ponatinib hydrochloride	Pregnancy-contraindicated	Contraindicated (Mandatory contraception)	62589
429	Trifluridine/Tipiracil hydrochloride combination	Pregnancy-contraindicated	Contraindicated (Mandatory contraception)	56666
429	Lenvatinib mesilate	Pregnancy-contraindicated	Contraindicated (Mandatory contraception)	50081
429	Ibrutinib	Pregnancy-contraindicated	Contraindicated (Mandatory contraception)	27150
429	Regorafenib hydrate	Pregnancy-contraindicated	Contraindicated (Mandatory contraception)	25976
429	Axitinib	Pregnancy-contraindicated	Contraindicated (Mandatory contraception)	19292
429	Cabozantinib malate	Use if benefit outweighs risk	Contraindicated (Mandatory contraception)	15761
429	Sunitinib malate	Pregnancy-contraindicated	Contraindicated (Mandatory contraception)	14147
429	Tirabrutinib hydrochloride	Use if benefit outweighs risk	Contraindicated (Mandatory contraception)	11702
429	Abiraterone acetate	No cautionary statement	No cautionary statement	<1000
429	Apalutamide	Use if benefit outweighs risk	No cautionary statement	<1000
429	Bicalutamide	No cautionary statement	No cautionary statement	<1000
429	Darolutamide	No cautionary statement	No cautionary statement	<1000

429	Enzalutamide	No cautionary statement	No cautionary statement	<1000
429	Flutamide	No cautionary statement	No cautionary statement	<1000
442	Bucillamine	Use if benefit outweighs risk	No cautionary statement	1575529
442	Auranofin	Pregnancy-contraindicated	No cautionary statement	6762
449	Fexofenadine hydrochloride	Use if benefit outweighs risk	No cautionary statement	160485726
449	Montelukast sodium	Use if benefit outweighs risk	No cautionary statement	120252240
449	Olopatadine hydrochloride	Use if benefit outweighs risk	No cautionary statement	119388767
449	Bepotastine besilate	Use if benefit outweighs risk	No cautionary statement	110368551
449	Bilastine	Use if benefit outweighs risk	No cautionary statement	88612694
449	Levocetirizine hydrochloride	Use if benefit outweighs risk	No cautionary statement	75078479
449	Pranlukast hydrate	Use if benefit outweighs risk	No cautionary statement	62819523
449	Rupatadine fumarate	Use if benefit outweighs risk	No cautionary statement	50084391
449	Desloratadine	Use if benefit outweighs risk	No cautionary statement	43259476
449	Fexofenadine hydrochloride/Pseudoephedrine hydrochloride combination	Use if benefit outweighs risk	No cautionary statement	34541562
449	Epinastine hydrochloride	Use if benefit outweighs risk	No cautionary statement	26063163
449	Loratadine	Use if benefit outweighs risk	No cautionary statement	23598125
449	Cedacure (Japanese cedar pollen sublingual tablets)	Use if benefit outweighs risk	No cautionary statement	13195466
449	Tranilast	Pregnancy-contraindicated	No cautionary statement	12762248
449	Cetirizine hydrochloride	Use if benefit outweighs risk	No cautionary statement	10416297
449	Suplatast tosilate	Use if benefit outweighs risk	No cautionary statement	10175930
449	Ebastine	Use if benefit outweighs risk	No cautionary statement	7212779
449	Miticure (house dust mite sublingual tablets)	Use if benefit outweighs risk	No cautionary statement	5467730
449	Ramatroban	Use if benefit outweighs risk	No cautionary statement	3901251
449	Emedastine fumarate	Use if benefit outweighs risk	No cautionary statement	2550712
449	Azelastine hydrochloride	Use if benefit outweighs risk	No cautionary statement	2199571
449	Pemirolast potassium	Pregnancy-contraindicated	No cautionary statement	1637401

449	Ketotifen fumarate	Use if benefit outweighs risk	No cautionary statement	1536772
449	Oxatomide	Pregnancy-contraindicated	No cautionary statement	965363
449	Ibudilast	Use if benefit outweighs risk	No cautionary statement	723537
449	Acitea (house dust mite sublingual tablets)	Use if benefit outweighs risk	No cautionary statement	370391
449	Ozagrel hydrochloride hydrate	Use if benefit outweighs risk	No cautionary statement	237644
449	Seratrodast	Use if benefit outweighs risk	No cautionary statement	64515
617	Amphotericin B	Use if benefit outweighs risk	No cautionary statement	507829
617	Voriconazole	Pregnancy-contraindicated	No cautionary statement	262859
617	Posaconazole	Use if benefit outweighs risk	Contraindicated (Mandatory contraception)	71638
619	Bonosap pack (Vonoprazan fumarate/Amoxicillin hydrate/Clarithromycin)	Use if benefit outweighs risk	No cautionary statement	494704
619	Rifaximin	Use if benefit outweighs risk	No cautionary statement	419833
619	Bonopion pack (Vonoprazan fumarate/Amoxicillin hydrate/Metronidazole combination)	Use if benefit outweighs risk	No cautionary statement	55671
619	Ampicillin hydrate/Cloxacillin sodium hydrate combination	Use if benefit outweighs risk	No cautionary statement	46183
619	Rabecure pack (Rabeprazole sodium/Amoxicillin hydrate/Clarithromycin)	Use if benefit outweighs risk	No cautionary statement	12005
619	Rabefine pack (Rabeprazole sodium/Amoxicillin hydrate/Clarithromycin)	Pregnancy-contraindicated	No cautionary statement	1211
621	Salazosulfapyridine	Use if benefit outweighs risk	No cautionary statement	12782677
621	Sulfamethoxazole/Trimethoprim combination	Pregnancy-contraindicated	No cautionary statement	<1000
622	Ethambutol hydrochloride	Use if benefit outweighs risk	No cautionary statement	884291
622	Isoniazid	Use if benefit outweighs risk	No cautionary statement	770907
622	Pyrazinamide	Use if benefit outweighs risk	No cautionary statement	45446
622	Delamanid	Contraindicated	No cautionary statement	17244
622	Ethionamide	Use if benefit outweighs risk	No cautionary statement	7353
622	Aluminoparaaminosalicylate calcium hydrate	Use if benefit outweighs risk	No cautionary statement	3465
622	Isoniazid sodium methanesulfonate hydrate	Use if benefit outweighs risk	No cautionary statement	3429

622	Powdered glycyrrhiza/Powdered senna leaf/Powdered fennel/Sulfur/White soft sugar combination	Use if benefit outweighs risk	No cautionary statement	3196
622	Calcium paraaminosalicylate hydrate	Use if benefit outweighs risk	No cautionary statement	2709
624	Levofloxacin hydrate	Pregnancy-contraindicated	No cautionary statement	6459596
624	Garenoxacin mesilate hydrate	Pregnancy-contraindicated	No cautionary statement	4224105
624	Tosufloxacin tosilate hydrate	Pregnancy-contraindicated	No cautionary statement	2867593
624	Lascufloxacin hydrochloride	Pregnancy-contraindicated	No cautionary statement	2213210
624	Sitafloxacin hydrate	Pregnancy-contraindicated	No cautionary statement	2171244
624	Ciprofloxacin	Pregnancy-contraindicated	No cautionary statement	419453
624	Prulifloxacin	Pregnancy-contraindicated	No cautionary statement	384443
624	Ofloxacin	Pregnancy-contraindicated	No cautionary statement	185757
624	Norfloxacin	Pregnancy-contraindicated	No cautionary statement	162747
624	Moxifloxacin hydrochloride	Pregnancy-contraindicated	No cautionary statement	52091
624	Lomefloxacin hydrochloride	Pregnancy-contraindicated	No cautionary statement	33882
624	Linezolid	Use if benefit outweighs risk	No cautionary statement	<1000
624	Tedizolid phosphate	Use if benefit outweighs risk	No cautionary statement	<1000
625	Valaciclovir hydrochloride	Use if benefit outweighs risk	No cautionary statement	8507932
625	Famciclovir	Use if benefit outweighs risk	No cautionary statement	2115848
625	Oseltamivir phosphate	Use if benefit outweighs risk	No cautionary statement	1402389
625	Tenofovir alafenamide fumarate	Use if benefit outweighs risk	No cautionary statement	1335983
625	Entecavir hydrate	Use if benefit outweighs risk	Contraindicated (Mandatory contraception)	753459
625	Molnupiravir	Pregnancy-contraindicated	Contraindicated (Mandatory contraception)	720264
625	Amenamevir	Use if benefit outweighs risk	No cautionary statement	702668
625	Aciclovir	Use if benefit outweighs risk	No cautionary statement	608965
625	Baloxavir marboxil	Use if benefit outweighs risk	No cautionary statement	200743
625	Tenofovir disoproxil fumarate	Use if benefit outweighs risk	No cautionary statement	106920

625	Glecaprevir hydrate/Pibrentasvir combination	Use if benefit outweighs risk	No cautionary statement	58743
625	Bictegravir sodium/Emtricitabine/Tenofovir alafenamide fumarate combination	Use if benefit outweighs risk	No cautionary statement	55543
625	Valganciclovir hydrochloride	Pregnancy-contraindicated	Contraindicated (Mandatory contraception)	53948
625	Raltegravir potassium	Use if benefit outweighs risk	No cautionary statement	48856
625	Emtricitabine/Tenofovir Alafenamide fumarate combination	Use if benefit outweighs risk	No cautionary statement	37845
625	Dolutegravir sodium	Use if benefit outweighs risk	No cautionary statement	25240
625	Dolutegravir sodium/Lamivudine combination	Use if benefit outweighs risk	No cautionary statement	22936
625	Letermovir	Use if benefit outweighs risk	Contraindicated (Mandatory contraception)	13667
625	Lamivudine (indication: hepatitis B virus infection)	Use if benefit outweighs risk	No cautionary statement	8918
625	Elvitegravir/Cobicistat/Emtricitabine/Tenofovir/Alafenamide fumarate combination	Use if benefit outweighs risk	No cautionary statement	5945
625	Dolutegravir sodium/Abacavir sulfate/Lamivudine combination	Use if benefit outweighs risk	No cautionary statement	5914
625	Darunavir Ethanolate/Cobicistat/Emtricitabine/Tenofovir Alafenamide fumarate combination	Use if benefit outweighs risk	No cautionary statement	5251
625	Emtricitabine/Tenofovir disoproxil fumarate combination	Use if benefit outweighs risk	No cautionary statement	4080
625	Rilpivirine hydrochloride/Tenofovir Alafenamide fumarate/Emtricitabine combination	Use if benefit outweighs risk	No cautionary statement	3890
625	Lamivudine/Abacavir sulfate combination	Use if benefit outweighs risk	No cautionary statement	3536
625	Lopinavir/Ritonavir combination	Use if benefit outweighs risk	No cautionary statement	3016
625	Doravirine	Use if benefit outweighs risk	No cautionary statement	2740
625	Darunavir Ethanolate/Cobicistat combination	Use if benefit outweighs risk	No cautionary statement	2692
625	Lamivudine (indication: HIV infection)	Use if benefit outweighs risk	No cautionary statement	2220
625	Ledipasvir acetonate/Sofosbuvir combination	Use if benefit outweighs risk	No cautionary statement	1694
625	Ritonavir	Use if benefit outweighs risk	No cautionary statement	1320
625	Abacavir sulfate	Use if benefit outweighs risk	No cautionary statement	<1000
625	Adefovir dipivoxil	Use if benefit outweighs risk	No cautionary statement	<1000
625	Dolutegravir sodium/Rilpivirine hydrochloride combination	Use if benefit outweighs risk	No cautionary statement	<1000

625	Efavirenz	Use if benefit outweighs risk	Contraindicated (Mandatory contraception)	<1000
625	Etravirine	Use if benefit outweighs risk	No cautionary statement	<1000
625	Rilpivirine hydrochloride	Use if benefit outweighs risk	No cautionary statement	<1000
629	Trimethoprim/Sulfamethoxazole combination	Pregnancy-contraindicated	No cautionary statement	4891578
629	Itraconazole	Pregnancy-contraindicated	Contraindicated (Mandatory contraception)	2046452
629	Terbinafine hydrochloride	Use if benefit outweighs risk	No cautionary statement	982203
629	Fosravuconazole L-lysine ethanolate	Pregnancy-contraindicated	Contraindicated (Mandatory contraception)	687352
629	Miconazole	Pregnancy-contraindicated	No cautionary statement	602713
629	Atovaquone	Use if benefit outweighs risk	No cautionary statement	444267
629	Fluconazole	Pregnancy-contraindicated	No cautionary statement	423983
629	Flucytosine	Pregnancy-contraindicated	No cautionary statement	3974
641	Metronidazole	Use if benefit outweighs risk	No cautionary statement	1242557
641	Spiramycin	No cautionary statement	No cautionary statement	325722
641	Tinidazole	Pregnancy-contraindicated	No cautionary statement	4291
641	Artemether/Lumefantrine combination	Pregnancy-contraindicated	Contraindicated (Mandatory contraception)	<1000
641	Atovaquone/Proguanil hydrochloride combination	Use if benefit outweighs risk	No cautionary statement	<1000
641	Mefloquine hydrochloride	Pregnancy-contraindicated	Contraindicated (Mandatory contraception)	<1000
641	Paromomycin sulfate	Use if benefit outweighs risk	Contraindicated (Mandatory contraception)	<1000
641	Primaquine phosphate	Pregnancy-contraindicated	Contraindicated (Mandatory contraception)	<1000
642	Ivermectin	Use if benefit outweighs risk	No cautionary statement	40180
642	Albendazole	Pregnancy-contraindicated	Contraindicated (Mandatory contraception)	3856
642	Diethylcarbamazine citrate	Use if benefit outweighs risk	No cautionary statement	<1000

642	Mebendazole	Pregnancy-contraindicated	No cautionary statement	<1000
642	Praziquantel	Use if benefit outweighs risk	No cautionary statement	<1000
642	Pyrantel pamoate	Use if benefit outweighs risk	No cautionary statement	<1000
642	Thymol	No cautionary statement	No cautionary statement	<1000
